# Supplementary material for: Association between early child development trajectories and adult cognitive function in a 50-year longitudinal study in Guatemala
Source: BMJ Open. 2021 Jun 30;11(6):e044966. doi: 10.1136/bmjopen-2020-044966 (PMC8246364; doi:10.1136/bmjopen-2020-044966)
Supplement: Supplementary data [file bmjopen-2020-044966supp001.pdf]

Supplemental Table 1: Sample size of childhood cognitive development and adult Raven Progressive Matrices assessments by birth year in the INCAP Nutrition Supplementation Trial Longitudinal Study (n=2392)

Year of birth	Infant development scales			Preschool Series					≥ 3 child assessments	≥ 1 child assessments	≥ 1 adult assessments	≥ 1 adult and >1 child assessments
	6 months	15 months	24 months	3 years	4 years	5 years	6 years	7 years				
1962	0	0	0	0	0	0	0	47	0	47	60	39
1963	0	0	0	0	0	0	66	86	0	89	80	69
1964	0	0	0	0	0	53	73	78	47	83	63	54
1965	0	0	0	0	63	91	96	97	88	105	97	86
1966	0	0	0	66	98	100	104	101	99	116	113	99
1967	0	1	34	78	92	94	92	96	92	110	112	94
1968	12	44	57	93	95	100	95	94	97	117	114	96
1969	76	84	84	113	118	120	122	122	125	145	146	126
1970	97	95	92	112	113	110	117	18	118	141	143	124
1971	113	116	106	125	128	131	26	0	130	156	146	126
1972	99	114	93	124	129	34	0	0	117	152	168	136
1973	0	15	0	25	24	0	0	0	14	26	133	23
1974	0	0	0	0	0	0	0	0	0	0	148	0
1975	0	0	0	0	0	0	0	0	0	0	133	0
1976	0	0	0	0	0	0	0	0	0	0	135	0
1977	0	0	0	0	0	0	0	0	**0**	0	84	0
**Total**	**397**	**469**	**466**	**736**	**860**	**833**	**791**	**739**	**927**	**1287**	**1875**	**1072**
