# Supplementary material for: Association between early child development trajectories and adult cognitive function in a 50-year longitudinal study in Guatemala
Source: BMJ Open. 2021 Jun 30;11(6):e044966. doi: 10.1136/bmjopen-2020-044966 (PMC8246364; doi:10.1136/bmjopen-2020-044966)
Supplement: Author's manuscript [file bmjopen-2020-044966.draft_revisions.pdf]

BMJ Open

BMJ Open is committed to open peer review. As part of this commitment we make the peer review history of every article we publish publicly available.

When an article is published we post the peer reviewers' comments and the authors' responses online. We also post the versions of the paper that were used during peer review. These are the versions that the peer review comments apply to.

The versions of the paper that follow are the versions that were submitted during the peer review process. They are not the versions of record or the final published versions. They should not be cited or distributed as the published version of this manuscript.

BMJ Open is an open access journal and the full, final, typeset and author-corrected version of record of the manuscript is available on our site with no access controls, subscription charges or pay-per-view fees (http://bmjopen.bmj.com).

If you have any questions on BMJ Open's open peer review process please email info.bmjopen@bmj.com

BMJ Open

Trajectories of child development predict adult cognitive function: a 50-year prospective study in Guatemala

Journal:	*BMJ Open*
Manuscript ID	bmjopen-2020-044966
Article Type:	Original research
Date Submitted by the Author:	25-Sep-2020
Complete List of Authors:	Gonzalez Casanova, Ines; Indiana University Bloomington, Applied Health Science; Emory University,<br>DiGirolamo, Ann ; Georgia State University<br>Kroker-Lobos, Maria; INCAP<br>Ochaeta, Laura; INCAP<br>Ramirez-Zea, Manuel; INCAP<br>Martorell, Reynaldo; Emory University<br>Stein, Aryeh D.; Emory University,
Keywords:	EPIDEMIOLOGY, International health services < HEALTH SERVICES ADMINISTRATION & MANAGEMENT, MENTAL HEALTH, Developmental neurology & neurodisability < PAEDIATRICS, PUBLIC HEALTH

SCHOLARONE™
Manuscripts

I, the Submitting Author has the right to grant and does grant on behalf of all authors of the Work (as defined in the below author licence), an exclusive licence and/or a non-exclusive licence for contributions from authors who are: i) UK Crown employees; ii) where BMJ has agreed a CC-BY licence shall apply, and/or iii) in accordance with the terms applicable for US Federal Government officers or employees acting as part of their official duties; on a worldwide, perpetual, irrevocable, royalty-free basis to BMJ Publishing Group Ltd ("BMJ") its licensees and where the relevant Journal is co-owned by BMJ to the co-owners of the Journal, to publish the Work in this journal and any other BMJ products and to exploit all rights, as set out in our licence.

The Submitting Author accepts and understands that any supply made under these terms is made by BMJ to the Submitting Author unless you are acting as an employee on behalf of your employer or a postgraduate student of an affiliated institution which is paying any applicable article publishing charge ("APC") for Open Access articles. Where the Submitting Author wishes to make the Work available on an Open Access basis (and intends to pay the relevant APC), the terms of reuse of such Open Access shall be governed by a Creative Commons licence – details of these licences and which Creative Commons licence will apply to this Work are set out in our licence referred to above.

Other than as permitted in any relevant BMJ Author's Self Archiving Policies, I confirm this Work has not been accepted for publication elsewhere, is not being considered for publication elsewhere and does not duplicate material already published. I confirm all authors consent to publication of this Work and authorise the granting of this licence.

For peer review only

Title: Trajectories of child development predict adult cognitive function: a 50-year prospective study in Guatemala

Authors: Ines Gonzalez-Casanova[1,2], Ann DiGirolamo[3], Maria F. Kroker-Lobos[4], Laura Ochaeta[4], Manuel Ramirez-Zea[4], Reynaldo Martorell[2], Aryeh D. Stein[2]*,

1. Department of Applied Health Sciences, Indiana University Bloomington School of Public Health, IN, USA, 2. Hubert Department of Global Health, Rollins School of Public Health, Emory University, Atlanta, GA, USA, 3. Georgia Health Policy Center, Andrew Young School of Policy Studies, Georgia State University, Atlanta, Georgia, USA, 4. INCAP Research Center for the Prevention of Chronic Diseases, Institute of Nutrition of Central America and Panama, Guatemala City, Guatemala

***Corresponding Author**
Aryeh D. Stein, PhD
email: aryeh.stein@emory.edu

For peer review only

Article Summary

Strengths and limitations of this study

- Repeated measures of cognitive function from 6 months to 55 years of age
- Birth cohort with 50 years of follow-up from Guatemala, a transitioning country
- Longitudinal analysis using latent class growth analysis
- There was missing information from some participants
- Results are consistent with what has been reported in studies from high-income countries

Abstract

Background: in order to develop effective interventions to improve life-course cognition, it is important to understand the relationship between early childhood development and adult cognitive functioning in diverse populations. Few studies have used longitudinal data to study the development of cognition over the life course in low- and middle- income countries.
Methods: We analyzed data from the INCAP Nutrition Supplementation Trial Longitudinal Study in Guatemala. Cognition was assessed at eight different time-points between 6 months and 7 years. We derived childhood development trajectories using latent class growth analysis. We assessed predictors of the trajectories using ordinal logistic regression, and associations between childhood trajectories and adult non-verbal intelligence and literacy at age 11 to 55 years (mean ± SD =42.7 ± 6.4 years) using generalized linear models.
Results: Among 927 participants, we identified three trajectories of cognitive development (high: n=214, average: n=583, low: n=130). Participants whose mothers were taller [proportional log odds (PO)=0.03 95%CI=(0.01, 0.06)], had more years of schooling [PO=0.15 95%CI = (0.06, 0.25)], or lived in households with higher socioeconomic scores [PO = 0.19 95%CI=(0.09, 0.29)] were more likely to follow higher trajectories.
Conclusions: In this sample from Guatemala, cognitive development trajectories from 6 months through 7 years were associated with adult non-verbal intelligence and literacy. These findings provide evidence of tracking of cognition over-time in a transitioning low-income setting.

For peer review only - http://bmjopen.bmj.com/site/about/guidelines.xhtml

INTRODUCTION

In low- and middle- income (LMIC) countries, 43% of children younger than five years were considered at risk of poor development in 2010 using stunting and poverty as proxies. [1] Early childhood development and childhood intellectual functioning predict survival, lifelong health, and human capital; [2-5] hence, assuring that children worldwide reach their full cognitive developmental potential is central to the sustainable development agenda. [1]

This has led to a widespread call to implement effective interventions that improve early cognitive development.[1, 6, 7] However, the complex nature of the known predictors of early development and challenges assessing development and intelligence across time in diverse populations have complicated the targeting and evaluation of interventions to improve life-course cognitive functioning , especially in LMIC.[8, 9]

Understanding how cognitive functioning tracks across time in diverse settings is an important step towards identifying effective approaches to improve childhood development; however, information from LMIC is limited. Considering that LMIC have the greatest proportion of children at risk and are primary targets for interventions and that the first years of life are the most important period for brain growth and differentiation, it is important to address these gaps in knowledge from LMIC. Following a life-course approach and taking advantage of a long-running cohort from Guatemala, [10] the objective of this study was to assess childhood cognitive developmental trajectories during the first seven years of life, to identify predictors of these trajectories, and to estimate the associations of these trajectories with adult cognitive functioning.

For peer review only - http://bmjopen.bmj.com/site/about/guidelines.xhtml

METHODS

Longitudinal study

We used data from the INCAP Nutrition Supplementation Trial Longitudinal Study [10]. The study was originally conceived as a trial to test the hypothesis that providing a protein-energy supplement during the preschool years would improve mental development. The specific methods for the original trial have been described extensively; [10-13] briefly, four villages in Guatemala were assigned to receive either a protein-energy gruel (Atole) or a protein-free sugary drink (Fresco). The supplements were provided to all residents in the study villages in a central location twice daily from 1969 through 1977; intakes were recorded for pregnant and lactating women and children under the age of 7 years. In total, 2392 children were enrolled in the original study because they were either younger than seven years at the beginning of the trial or were born during its implementation; hence, participants in this cohort were born between 1962 and 1977 in the study villages.[14] Follow-up studies have been conducted in 1988-99, 2002-04, and 2015-17. [10, 15, 16]

Participant and Public Involvement

Participants and community were not involved in the initial design of the intervention or outcome selection, but over the years the study team provided feedback to the communities about the study and its findings.

Analytical Sample and Data for this Analysis

For peer review only - http://bmjopen.bmj.com/site/about/guidelines.xhtml

We included all participants with at least three measures of cognitive development out of the eight potentially available. This is a conservative approach as latent class growth analysis can handle some participants having as few as one measurement. [17] For the analysis testing associations with adult intelligence and literacy, we excluded participants missing information on these measurements. Information on sociodemographic predictors such as parents' years of schooling, household socioeconomic status, and distance to the health center was extracted from data collected during the original supplementation study (between 1969 and 1977).

Measures

Cognitive Development Assessments

The cognitive function assessments for infants and preschool children were specifically developed for the study using items from different age-appropriate tests.[18]

Infant battery (administered at child ages 6, 15 and 24 months)-

This set of instruments was administered within a two-week window of infants turning 6, 15, and 24 months. It was developed as a compendium of relevant items from validated infant scales including the Bayley Scales of Infant Development [19], the Gesell and Amatruda's Developmental Diagnosis [20], the Cattell Infant's Intelligence Scales [21], and Merrill-Palmer Standards of Physical and Mental Growth [22]. The selected items were translated into Spanish and adapted for the Guatemalan context by trained psychologists and psychiatrists. The Composite Infant Score (CIS) summarizes 44 items derived from the previously mentioned scales.

Preschool battery (administered at child ages 3, 4, 5, 6 and 7 years) -

For peer review only - http://bmjopen.bmj.com/site/about/guidelines.xhtml

A set of 22 tests was applied within a month of the participants' fifth, sixth and seventh birthday, and a shorter set of 10 tests was applied within a month of children's third and fourth birthdays. The preschool battery measured various dimensions of cognition (e.g. verbal ability, general knowledge, quantitative skills and memory). These scales have previously been analyzed by factor analysis, resulting in a general and a memory factor, which were deemed reliable and valid predictors of later development.[23]

Raven's Progressive Matrices (RPM) –

The RPM was applied during the 1988-1989, 2004-2005, and 2015-2017 data collection waves, when participants were between 11-27, 22-43, and 37-55 years old, respectively. The RPM is a non-verbal test of intellectual functioning and abstract reasoning that includes a series of pattern-matching exercises that get progressively more complex.[24] The RPM traditionally consists of five sets of 12 items each, however only the first three sets were applied, because in our pilot work we found that few participants could process further than that. [14] The score was calculated by summing the number of correct answers. The RPM has good test-retest reliability (intra-class correlation coefficient, 0.87) and internal consistency, as well as construct validity in this Guatemalan population.[14]

Adult literacy and reading comprehension

InterAmerican Reading Series (Serie Interamericana) –

This test was administered in 2004-2005 and 2015-2017. To complete this test, those who were unable to read a test sentence from the local paper or had not completed primary school, received the pre-screening test questions where scores could range between zero and five. Those who passed the screening test (by answering the five questions correctly) or had at least a secondary

For peer review only - http://bmjopen.bmj.com/site/about/guidelines.xhtml

school education started with a score of 5 which was then added to the InterAmerican reading and writing series composite score. This approach has been found to have adequate test-retest reliability and internal consistency in this population [25].

Statistical Analysis

Summarizing child cognitive data into trajectories

In order to make the scores comparable over time, we first converted the individual scores for mental development on the CIS at 6, 15 and 24 months, and the preschool series composite score at 3, 4, 5, 6, and 7 years into z-scores relative to the same population and age. We assessed Spearman pairwise correlations between all the available childhood cognitive development measurements during the first seven years and RPM measurements stratified by age. We then used LCGA [17] to cluster participants into developmental trajectories during the first seven years based on the age-adjusted ranked scores. LGCA classifies individuals based on their cognitive development at different ages into classes or trajectories with distinct intercepts and slopes, thereby capturing the heterogeneity within a sample. Because of the way the original study was designed, in order to account for potentially systematically missing information by birth year (supplemental table 1), a sensitivity analysis was conducted by including children with at least one CIS and at least one preschool measurement. All LCGA were adjusted for sex and for maternal ID (to account for siblings in the sample). The LCGA analysis was performed using M-Plus (Version 7.0, Los Angeles, CA).

Assessing predictors of child cognitive development trajectories:

We assessed if family-level variables predicted the trajectories using ordinal logistic regression. Models were developed based on theory and available data on the household sociodemographic

For peer review only - http://bmjopen.bmj.com/site/about/guidelines.xhtml

determinants of child development, which included SES score, parents' education and height, family size (number of people living in the same household), and distance from the supplementation center. All models were adjusted for type of supplement (operationalized as village of residence) and age at exposure to the supplement (first 1000 days vs. not within the first 1000 days). Multiple imputation in SAS 9.4 was conducted to account for item-specific missing values in the predictors. We used PROC MI to generate twenty imputed datasets using fully conditional specification. Ordinal logistic regression was then conducted for each of the twenty imputed datasets and the estimates were pooled using PROC MIANALYZE.

Testing associations between child development measurements, trajectories and adult outcomes

We used the last available RPM measure (mean age ± SD = 42.7 ± 6.4). For the literacy score, we used the last score recorded. We used ANOVA to assess mean RPM and literacy score differences by trajectory and by 7-year tertiles as comparison.

Data management and analysis were conducted using SAS 9.4 (SAS Institute Inc., Cary, NC, USA) unless otherwise noted.

RESULTS

The sample for the trajectory analysis and early predictors included 927 participants with at least three measurements of cognitive development during childhood (Supplemental Table 1). Sample sizes for testing associations between trajectory membership and adult cognitive functioning varied; in total, 823 participants could be classified into childhood trajectories and had at least one RPM measurement (Figure 1).

[Figure 1- Sample flowchart for cognitive development trajectory analysis in the INCAP Nutrition Supplementation Trial Longitudinal Study in Guatemala]

For peer review only - http://bmjopen.bmj.com/site/about/guidelines.xhtml

In the LCGA, based on model fit indexes, the sample membership for each trajectory and interpretability of the resulting classes, we identified three trajectories which we labeled low, average, and high (Figure 2). A sensitivity analysis including only children with at least one infant and one preschool measurement (n=577) also resulted in three similar trajectories. Differences among the three trajectories were apparent already at 6 months and became larger as the children grew older.

[Figure 2: Cognitive development trajectories from 6 months to 7 years (84 months) of age (n=927)]

The weakest Spearman correlations with the adult RPM assessments were with the infant battery at 6 and 15 months and with the preschool battery at 3 years, followed by the infant battery at 2 years and the preschool battery at 4 years. All the correlations between the 5-, 6-, and 7-year measurements, the trajectories, and the adult RPM were in the moderate range. The strongest correlations between childhood assessments and the RPM were with the preschool battery at 6 years, which ranged between 0.40 (at 11-20 and 21-36 years) and 0.43 (at 37-55 years). The 7-year preschool battery had the strongest correlation with the RPM at ages 11-20 years; the trajectories had the weakest correlation with measurements at 11-20 years (0.30) but stronger correlations with the RPM at 21-36 years (0.35) and 37-55 years (0.38). The RPM at 11-20 years had a correlation of 0.54 with the RPM at 37 to 55 years (Table 1).

Participants in the high trajectory belonged to households that, on average, had higher socioeconomic status, had mothers and fathers with more years of schooling, and had taller mothers, relative to those in the average trajectory. Conversely, the low trajectory had, on average, lower household socioeconomic status, their parents had fewer years of schooling and their mothers were shorter than those in the average trajectory (Table 2).

For peer review only - http://bmjopen.bmj.com/site/about/guidelines.xhtml

In proportional odds cumulative logit models, maternal and paternal education and height, and SES were positively associated with the trajectories, while family size was inversely associated with the trajectories (Table 3). After adjusting for all other covariates, the positive association with maternal schooling, maternal height and household SES remained, as did the inverse association with family size.

Child development trajectories were associated with adult RPM and literacy scores (Table 4). The mean and standard deviation scores for the latest RPM were 16.0 and 6.3, respectively; on average, participants in the low childhood cognitive development trajectory scored 5.6 fewer points on the RPM compared to those in the high trajectory (a difference of 0.93 standard deviations of the mean). The mean and standard deviation scores for the latest literacy scores were 49.8 and 28.6, respectively. The mean difference between the low and high trajectory for literacy scores was 29.6 (which represents 1.03 standard deviations of the mean). The 7-year cognitive development score tertiles were also associated with RPM and literacy scores in adulthood. Those in the highest 7-year tertile scored on average 4.9 points higher on the RPM and 25.8 points higher on the literacy test compared to those in the lowest tertile.

DISCUSSION

The primary aim of this study was to use a life-course approach to assess child development longitudinally in a longstanding cohort from Guatemala, which has 50 years of follow-up, and, in line with this approach, to identify predictors of early development and correlations with adult non-verbal intelligence and literacy. To our knowledge, this is the first study to assess life-course measures of cognition from early life through adulthood in a LMIC using a developmental trajectory approach, assessing relationships over time.

For peer review only - http://bmjopen.bmj.com/site/about/guidelines.xhtml

We identified three trajectories of childhood cognitive development through age seven years that diverge early and then remain parallel through the early school years. Consistent with this finding, early factors such as household SES and family size at birth, as well as maternal years of schooling and height were associated with trajectory membership, independently of other sociodemographic variables. These findings support early life as a critical period and highlight the importance of holistic interventions that address the complex interplay between sociodemographic factors, which ultimately can determine lifelong health advantages or vulnerabilities [26]. They also highlight the importance of investing in maternal education and nutrition within a context of a transitioning society to break the inter-generational transmission of inequality.

Results from this analysis, besides supporting early life as a critical period for the development of life-course cognition, also show a cumulative process where single time-point measurements at older ages (5-7 years) have stronger correlations with adult outcomes when compared to measurements conducted during infancy or early childhood (<4 years). The trajectory approach also provided more weight to the information from measurements conducted later in childhood. Taken together, results from this study highlight the importance of child cognitive development as a potential pathway linking early exposures and adult outcomes. This is important for the design and assessment of interventions and programs aiming to improve life-course development, health, human capital, as well as economic development.

We found mid-size correlations of 0.38 between cognitive development trajectories through seven years and non-verbal intelligence measured after 37 years of age. The trajectories created using repeated measurements of cognitive development by different instruments performed similarly to a single time point measurement at age 7 years of cognitive functioning in predicting

For peer review only - http://bmjopen.bmj.com/site/about/guidelines.xhtml

adult intelligence and literacy in this sample from Guatemala. The use of trajectories has the added advantage of maximizing the sample size, which can decrease bias. The stability of the trajectories decreases the need to conduct multiple tests that increase the risk of chance findings. Similarly, there is no consensus on the best age to assess the impact of interventions on development or to predict later cognitive outcomes as this might vary by setting and population;[1, 27, 28] trajectories allow utilizing all available information rather than relying on a single cross-sectional measurement. Another advantage of using LCGA was that we were able to interpret the trajectories and make qualitative inferences about the timing and processes of early cognition within sub-categories of this sample. For example, as mentioned before, the early separation of the trajectories highlights the importance of timing interventions during the prenatal period or early life.

The analysis of the correlation among the trajectories, individual childhood measurements, and adult outcomes also provided insights into the predictive value of individual time-measurements at different ages, which would not be evident with single measurements. In this instance, we showed higher correlations of individual child development measurements after 5 years with both the trajectories and adult non-verbal intelligence, when compared to earlier measurements. These results could be reflective of methodological challenges assessing development in younger children, [11, 29] as well as of cumulative processes and pathways that eventually lead to adult cognition.

Missing data is a limitation of this study. Less than half of the participants of the original study had three or more measurements of cognitive development and, due to the study design, most participants have missing data either at younger or older childhood measurements. However, to account for this, we conducted a sensitivity analysis including only those participants with at

For peer review only - http://bmjopen.bmj.com/site/about/guidelines.xhtml

least one measurement during infancy and at least one measurement during the preschool period and found similar trajectories. Another limitation with the study design was the large age-span of the cohort participants (i.e., 11 years), which forced us to include large age groups for adult measurements and complicated the study of specific periods during adulthood. A limitation of the LCGA approach is that the researcher selects the number of classes. We used model fit statistics and class size to select the number of classes but the process is empirical and not necessarily exempt from bias. Finally, there are additional factors that could have contributed to child cognitive development that were not measured in this study; for example, information on parents' intelligence or early childhood care and stimulation, among others factors, were not available.

Global estimates of the number of children not meeting their full developmental potential are currently based on poverty and stunting.[1, 30] Even though these two factors are associated with early childhood development, the use of proxies to assess cognition is limiting [9], and addressing this limitation is important in order to improve our ability to study the determinants of life-course cognition.[9] Results from this study contribute to further our understanding of cognitive functioning across the lifespan in LMIC. This cognitive development trajectory approach sets the basis for the study of life-course predictors of intelligence, which in turn can improve the design and targeting of interventions in diverse populations.

Funding: This study was funded by the Bill and Melinda Gates Foundation OPP1164115. The funder played no role in the design, implementation or interpretation of this study.

For peer review only - http://bmjopen.bmj.com/site/about/guidelines.xhtml

Table 1: Spearman pairwise correlations between cognitive development tests from 6 months through adulthood (n=1484 with at least 2 measurements of intelligence)

	r(n)[a]	Infant Battery			Preschool Battery						Raven Progressive Matrices		
		**6 mo**	**15 mo**	**24 mo**	**3 y**	**4 y**	**5 y**	**6 y**	**7 y**	**Trajectories**	**11-20 y**	**21-36 y**	**37-55 y**
**INFANT**	**6 mo**	*1.00* (397)	0.28 (312)	0.14 (293)	0.02 (344)	0.03 (342)	0.11 (277)	0.03 (180)	-0.11 (88)	0.14 (361)	0.08 (266)	0.11 (289)	0.08 (201)
	**15 mo**		*1.00* (469)	0.28 (312)	0.09 (425)	0.02 (425)	0.17 (332)	0.05 (222)	0.09 (129)	0.21 (447)	0.04 (325)	0.14 (345)	0.11 (236)
	**24 mo**			*1.00* (466)	0.36 (431)	0.09 (426)	0.16 (361)	0.28 (256)	0.25 (165)	0.39 (452)	0.11 (296)	0.21 (352)	0.18 (238)
**PRESCHOOL**	**3 y**				*1.00* (736)	0.29 (692)	0.29 (570)	0.26 (444)	0.24 (329)	0.45 (691)	0.03 (398)	0.09 (567)	0.03 (388)
	**4 y**					*1.00* (860)	0.41 (696)	0.33 (567)	0.31 (443)	0.53 (806)	0.13 (414)	0.21 (656)	0.23 (450)
	**5 y**						*1.00* (833)	0.60 (664)	0.54 (533)	0.71 (793)	0.35 (328)	0.34 (596)	0.32 (443)
	**6 y**							*1.00* (791)	0.66 (632)	0.77 (663)	0.43 (231)	0.40 (502)	0.40 (440)
	**7 y**								*1.00* (739)	0.76 (540)	0.45 (140)	0.34 (409)	0.35 (422)
	**Trajectories**[b]									1.00 (927)	0.30 (409)	0.36 (667)	0.38 (495)
**RAVEN**	**11-20y**										*1.00* (1297)	0.57 (753)	0.54 (523)
	**21-36y**											*1.00* (1452)	0.96 (855)
	**37-55y**												*1.00* (1163)

[a]Values are Spearman correlations between child z-scores and adult raw raven scores.
[b]The trajectories were coded low=0, average=1 and high=2 for this analysis.

For peer review only - http://bmjopen.bmj.com/site/about/guidelines.xhtml

Table 2: Sociodemographic and childhood characteristics by cognitive development trajectory

	**Low (130)**	**Average (583)**	**High (214)**[a]	**p-value**
Community-level predictor				
Large village (%)	56.1	55.3	55.0	0.97
Household-level predictors at enrollment				
Distance from supplementation center (Km)	2.7 ± 0.1	2.5 ± 0.1	2.4 ± 0.1	0.26
SES Score	-0.5 ± 0.1	-0.2 ± 0.1	0.2 ± 0.1	<0.001
Maternal schooling (years)	1.0 ± 0.1	1.2 ± 0.1	1.8 ± 1.1	<0.001
Paternal schooling (years)	1.3 ± 0.2	1.5 ± 0.1	2.0 ± 0.1	<0.001
Maternal height (cm)	148.0 ± 0.5	148.9 ± 0.2	150.0 ± 0.4	<0.001
Paternal height (cm)	160.1 ± 0.6	160.6 ± 0.3	161.6 ± 0.6	0.12
Family size	6.8 ± 0.2	6.7 ± 0.1	6.5 ± 0.2	0.34
Individual-level predictors				
Male (%)	52.6	51.6	54.2	0.83
Height 36 mo (cm)	83.5 ± 0.4	85.5 ± 0.2	86.7 ± 0.3	<0.001
Weight 36 mo (kg)	11.3 ± 0.2	11.8 ± 0.1	12.0 ±0.1	<0.001

[a]Trajectory analysis was adjusted by sex and age at measurement. Differences among groups were assessed using chi-square test for categorical variables and ANOVA for continuous variables.

For peer review only

Table 3: Proportional odds logistic regression of sociodemographic predictors and cognitive development trajectories (n=927)[a]

	Unadjusted		Adjusted[b]	
	**Estimate[a]**	**95% CI**	**Estimate[a]**	**95% CI**
SES Score	0.22	(0.13, 0.32)	0.19	(0.09, 0.29)
Family size (# of people)	-0.06	(-0.13, 0.00)	-0.08	(-0.16, -0.01)
Distance from supplementation center (Km)	-0.09	(-0.21, 0.03)	-0.03	(-0.16, 0.09)
Parents' schooling (years)				
Maternal	0.20	(0.12, 0.29)	0.15	(0.06, 0.25)
Paternal	0.12	(0.05, 0.18)	0.07	(0.00, 0.15)
Parents' height (cm)				
Maternal	0.04	(0.02, 0.07)	0.03	(0.01, 0.06)
Paternal	0.03	(0.00, 0.05)	0.01	(-0.02, 0.04)

[a] Estimates represent the proportional log odds of following a higher trajectory per unit increase in the predictor. Multiple imputation was used for item-specific missing values in the covariates. Trajectories were coded as low=0, average=1, high=2;

[b] Adjusted for all other covariates and for supplementation with Atole in the first 1000 days (sex and age at measurement were adjusted during the LCGA and thus were not included in this model).

For peer review only - http://bmjopen.bmj.com/site/about/guidelines.xhtml

Table 4: Associations between cognitive development trajectories, preschool series tertiles at 7 years, and adult literacy and non-verbal intelligence

	Trajectory [d]				7-years scores [e]			
	n	Low	Average	High	n	Low	Medium	High
Last available literacy score [a, c]	796	33.8 ± 2.6	48.2 ± 1.2	63.4 ± 2.0	604	33.6 ± 1.9	45.9 ± 1.9	59.4 ± 1.9
Last available non-verbal intelligence score[c]	823	13.8 ± 0.6	15.4 ± 0.3	19.4 ± 0.5	604	13.1 ± 0.4	14.9 ± 0.4	18.0 ± 0.4

[a]Measured by the InterAmerican Series;

[b]Measured by the Raven Progressive Matrices test;

[c]Mean ± standard deviation age at last measurement was 42.7 ± 6.4 years;

[d] Values are means and standard errors, mean differences by trajectory were assessed using ANOVA (all p-values <0.001);

[e]Values are means and standard errors by Preschool series 7-year score tertiles, mean differences by tertile were assessed using ANOVA (all p-values <0.001).

For peer review only - http://bmjopen.bmj.com/site/about/guidelines.xhtml

For peer review only

STATEMENTS

A. Contributorship statement

All authors contributed substantially to the conception of the work, acquisition, analysis and/or interpretation of the data as required by BMJ authorship requirements. IGC drafted the original manuscript and all authors reviewed it critically for intellectual content. All authors agree to be accountable for all aspects of the work reported in this manuscript.

B. Competing Interests

Authors declare no competing interests

C. Funding

This study was funded by the Bill and Melinda Gates Foundation (#OPP1164115). The funder was not involved in the design or interpretation of the results of this analysis.

D. Data Sharing statement

Data are available from the corresponding author upon written request.

REFERENCES

1 Black MM, Walker SP, Fernald LCH*, et al.* Early childhood development coming of age: science through the life course. *The Lancet* 2017;**389**:77-90.
2 Wraw C, Deary IJ, Gale CR*, et al.* Intelligence in youth and health at age 50. *Intelligence* 2015;**53**:23-32.
3 Jokela M, Elovainio M, Singh-Manoux A*, et al.* IQ, socioeconomic status, and early death: The US National Longitudinal Survey of Youth. *Psychosomatic medicine* 2009;**71**:322-8.
4 Calvin CM, Deary IJ, Fenton C*, et al.* Intelligence in youth and all-cause-mortality: systematic review with meta-analysis. *International journal of epidemiology* 2011;**40**:626-44.
5 McHutchison CA, Backhouse EV, Cvoro V*, et al.* Education, Socioeconomic Status, and Intelligence in Childhood and Stroke Risk in Later Life: A Meta-analysis. *Epidemiology (Cambridge, Mass)* 2017;**28**:608-18.
6 Richards M, Deary IJ. A life course approach to cognitive reserve: a model for cognitive aging and development? *Annals of neurology* 2005;**58**:617-22.
7 Kuh D, Network* tNDoAP. A Life Course Approach to Healthy Aging, Frailty, and Capability. *The Journals of Gerontology: Series A* 2007;**62**:717-21.
8 Sabanathan S, Wills B, Gladstone M. Child development assessment tools in low-income and middle-income countries: how can we use them more appropriately? *Archives of disease in childhood* 2015;**100**:482-8.
9 Richter LM, Black M, Britto P*, et al.* Early childhood development: an imperative for action and measurement at scale. *BMJ global health* 2019;**4**:e001302.

For peer review only - http://bmjopen.bmj.com/site/about/guidelines.xhtml

10 Stein AD, Melgar P, Hoddinott J, *et al.* Cohort Profile: the Institute of Nutrition of Central America and Panama (INCAP) Nutrition Trial Cohort Study. *International journal of epidemiology* 2008;**37**:716-20.
11 Hack M, Taylor HG, Drotar D, *et al.* Poor predictive validity of the Bayley Scales of Infant Development for cognitive function of extremely low birth weight children at school age. *Pediatrics* 2005;**116**:333-41.
12 Martorell R, Habicht JP, Rivera JA. History and design of the INCAP longitudinal study (1969-77) and its follow-up (1988-89). *J Nutr* 1995;**125**:1027s-41s.
13 Ramirez-Zea M, Mazariegos M. INCAP Longitudinal Study: 50 Years of History and Legacy. *Food and Nutrition Bulletin* 2020;**41**:S5-S7.
14 Pollitt E, Gorman KS, Engle PL, *et al.* Early Supplementary Feeding and Cognition: Effects over Two Decades. *Monographs of the Society for Research in Child Development* 1993;**58**:i-118.
15 Ford ND, Martorell R, Mehta NK, *et al.* Life-Course Body Mass Index Trajectories Are Predicted by Childhood Socioeconomic Status but Not Exposure to Improved Nutrition during the First 1000 Days after Conception in Guatemalan Adults. *The Journal of nutrition* 2016;**146**:2368-74.
16 Ford ND, Behrman JR, Hoddinott JF, *et al.* Exposure to improved nutrition from conception to age 2 years and adult cardiometabolic disease risk: a modelling study. *The Lancet Global Health* 2018;**6**:e875-e84.
17 Jung T, Wickrama KAS. An Introduction to Latent Class Growth Analysis and Growth Mixture Modeling. *Social and Personality Psychology Compass* 2008;**2**:302-17.
18 Engle PL, Fernandez PD. INCAP studies of malnutrition and cognitive behavior. *Food Nutr Bull* 2010;**31**:83-94.
19 Bayley N. *Bayley scales of infant development*. New York: The Psychological Corporation 1969.
20 Knobloch H, Stevens FM, Malone AF, *et al. Manual of developmental diagnosis : the administration and interpretation of the revised Gesell and Amatruda developmental and neurologic examination*. Hagerstown, Md.: Harper & Row 1980.
21 Cavanaugh MC, Cohen I, Dunphy D, *et al.* Prediction from the Cattell infant intelligence scale. *J Consult Psychol* 1957;**21**:33-7.
22 Merrill-Palmer Institute., Wilson CA. *The Merrill-Palmer standards of physical and mental growth*. Detroit 1930.
23 Pollitt E, Triana N. Stability, Predictive Validity, and Sensitivity of Mental and Motor Development Scales and Pre-School Cognitive Tests among Low-Income Children in Developing Countries. *Food and Nutrition Bulletin* 1999;**20**:45-52.
24 Raven J, Raven J. Raven Progressive Matrices. *Handbook of nonverbal assessment*. New York, NY, US: Kluwer Academic/Plenum Publishers 2003:223-37.
25 Stein AD, Wang M, DiGirolamo A, *et al.* Nutritional supplementation in early childhood, schooling, and intellectual functioning in adulthood: a prospective study in Guatemala. *Arch Pediatr Adolesc Med* 2008;**162**:612-8.
26 Maggi S, Irwin LJ, Siddiqi A, *et al.* The social determinants of early child development: An overview. *Journal of Paediatrics and Child Health* 2010;**46**:627-35.
27 Grantham-McGregor S, Cheung YB, Cueto S, *et al.* Developmental potential in the first 5 years for children in developing countries. *Lancet* 2007;**369**:60-70.

For peer review only

For peer review only - http://bmjopen.bmj.com/site/about/guidelines.xhtml

28 Prado EL, Dewey KG. Nutrition and brain development in early life. *Nutrition Reviews* 2014;**72**:267-84.
29 Aslin RN, Fiser J. Methodological challenges for understanding cognitive development in infants. *Trends in Cognitive Sciences* 2005;**9**:92-8.
30 Lu C, Black MM, Richter LM. Risk of poor development in young children in low-income and middle-income countries: an estimation and analysis at the global, regional, and country level. *The Lancet Global Health* 2016;**4**:e916-e22.

For peer review only

For peer review only - http://bmjopen.bmj.com/site/about/guidelines.xhtml

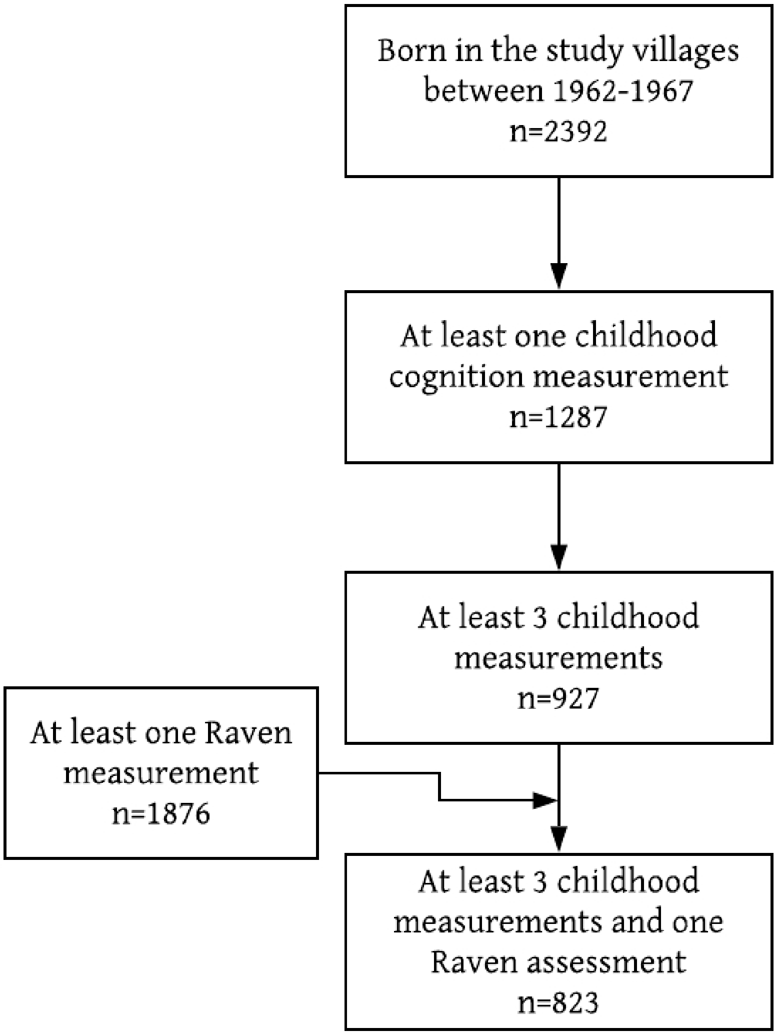

Figure 1- Sample flowchart for cognitive development trajectory analysis in the INCAP Nutrition Supplementation Trial Longitudinal Study in Guatemala

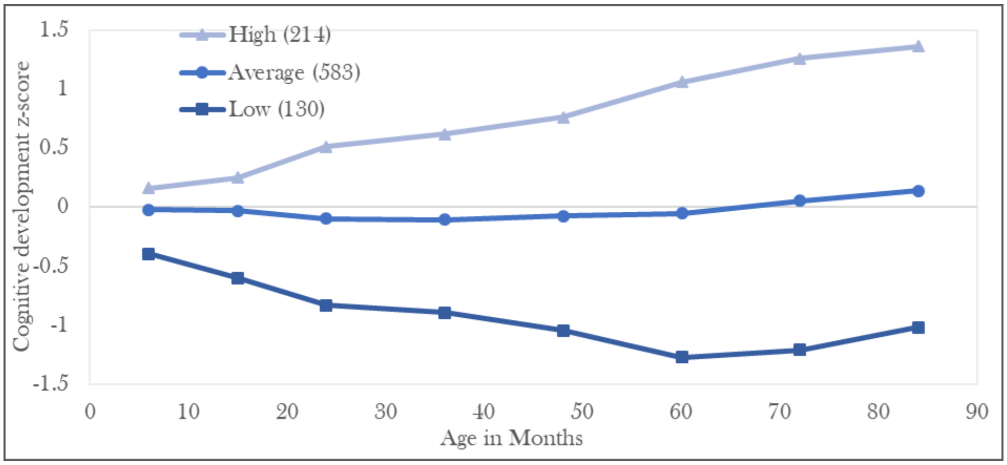

Figure 2: Cognitive development trajectories from 6 months to 7 years (84 months) of age (n=927)

Supplemental Table 1: Sample size of childhood cognitive development and adult Raven Progressive Matrices assessments by birth year in the INCAP Nutrition Supplementation Trial Longitudinal Study (n=2392)

Year of birth	Infant development scales			Preschool Series					≥ 3 child assessments	≥ 1 child assessments	≥ 1 adult assessments	≥ 1 adult and >1 child assessments
	6 months	15 months	24 months	3 years	4 years	5 years	6 years	7 years				
1962	0	0	0	0	0	0	0	47	0	47	60	39
1963	0	0	0	0	0	0	66	86	0	89	80	69
1964	0	0	0	0	0	53	73	78	47	83	63	54
1965	0	0	0	0	63	91	96	97	88	105	97	86
1966	0	0	0	66	98	100	104	101	99	116	113	99
1967	0	1	34	78	92	94	92	96	92	110	112	94
1968	12	44	57	93	95	100	95	94	97	117	114	96
1969	76	84	84	113	118	120	122	122	125	145	146	126
1970	97	95	92	112	113	110	117	18	118	141	143	124
1971	113	116	106	125	128	131	26	0	130	156	146	126
1972	99	114	93	124	129	34	0	0	117	152	168	136
1973	0	15	0	25	24	0	0	0	14	26	133	23
1974	0	0	0	0	0	0	0	0	0	0	148	0
1975	0	0	0	0	0	0	0	0	0	0	133	0
1976	0	0	0	0	0	0	0	0	0	0	135	0
1977	0	0	0	0	0	0	0	0	**0**	0	84	0
**Total**	**397**	**469**	**466**	**736**	**860**	**833**	**791**	**739**	**927**	**1287**	**1875**	**1072**

For peer review only

STROBE Statement—checklist of items that should be included in reports of observational studies

	Item No	Recommendation	Page No
**Title and abstract**	1	(*a*) Indicate the study's design with a commonly used term in the title or the abstract	1
		(*b*) Provide in the abstract an informative and balanced summary of what was done and what was found	1
**Introduction**			
Background/rationale	2	Explain the scientific background and rationale for the investigation being reported	2
Objectives	3	State specific objectives, including any prespecified hypotheses	2
**Methods**			
Study design	4	Present key elements of study design early in the paper	3
Setting	5	Describe the setting, locations, and relevant dates, including periods of recruitment, exposure, follow-up, and data collection	3
Participants	6	(*a*) *Cohort study*—Give the eligibility criteria, and the sources and methods of selection of participants. Describe methods of follow-up<br>*Case-control study*—Give the eligibility criteria, and the sources and methods of case ascertainment and control selection. Give the rationale for the choice of cases and controls<br>*Cross-sectional study*—Give the eligibility criteria, and the sources and methods of selection of participants	3
		(*b*) *Cohort study*—For matched studies, give matching criteria and number of exposed and unexposed<br>*Case-control study*—For matched studies, give matching criteria and the number of controls per case	NA
Variables	7	Clearly define all outcomes, exposures, predictors, potential confounders, and effect modifiers. Give diagnostic criteria, if applicable	4,5
Data sources/ measurement	8*	For each variable of interest, give sources of data and details of methods of assessment (measurement). Describe comparability of assessment methods if there is more than one group	*4,5*
Bias	9	Describe any efforts to address potential sources of bias	6
Study size	10	Explain how the study size was arrived at	4
Quantitative variables	11	Explain how quantitative variables were handled in the analyses. If applicable, describe which groupings were chosen and why	5,6
Statistical methods	12	(*a*) Describe all statistical methods, including those used to control for confounding	5,6
		(*b*) Describe any methods used to examine subgroups and interactions	5,6
		(*c*) Explain how missing data were addressed	5,6
		(*d*) *Cohort study*—If applicable, explain how loss to follow-up was addressed<br>*Case-control study*—If applicable, explain how matching of cases and controls was addressed<br>*Cross-sectional study*—If applicable, describe analytical methods taking account of sampling strategy	NA
		(*e*) Describe any sensitivity analyses	6

Continued on next page

For peer review only - http://bmjopen.bmj.com/site/about/guidelines.xhtml

**Results**			
Participants	13*	(a) Report numbers of individuals at each stage of study—eg numbers potentially eligible, examined for eligibility, confirmed eligible, included in the study, completing follow-up, and analysed	7, Figure 1, Supplemental table 1
		(b) Give reasons for non-participation at each stage	Figure 1
		(c) Consider use of a flow diagram	Figure 1
Descriptive data	14*	(a) Give characteristics of study participants (eg demographic, clinical, social) and information on exposures and potential confounders	7, Table 2
		(b) Indicate number of participants with missing data for each variable of interest	Table 1
		(c) *Cohort study*—Summarise follow-up time (eg, average and total amount)	NA
Outcome data	15*	*Cohort study*—Report numbers of outcome events or summary measures over time	*NA*
		Case-control study—Report numbers in each exposure category, or summary measures of exposure	*NA*
		Cross-sectional study—Report numbers of outcome events or summary measures	*NA*
Main results	16	(*a*) Give unadjusted estimates and, if applicable, confounder-adjusted estimates and their precision (eg, 95% confidence interval). Make clear which confounders were adjusted for and why they were included	8, Table 2, Table 3
		(*b*) Report category boundaries when continuous variables were categorized	NA
		(*c*) If relevant, consider translating estimates of relative risk into absolute risk for a meaningful time period	NA
Other analyses	17	Report other analyses done—eg analyses of subgroups and interactions, and sensitivity analyses	7,8
**Discussion**			
Key results	18	Summarise key results with reference to study objectives	9
Limitations	19	Discuss limitations of the study, taking into account sources of potential bias or imprecision. Discuss both direction and magnitude of any potential bias	11
Interpretation	20	Give a cautious overall interpretation of results considering objectives, limitations, multiplicity of analyses, results from similar studies, and other relevant evidence	9-11
Generalisability	21	Discuss the generalisability (external validity) of the study results	12
**Other information**			
Funding	22	Give the source of funding and the role of the funders for the present study and, if applicable, for the original study on which the present article is based	12

*Give information separately for cases and controls in case-control studies and, if applicable, for exposed and unexposed groups in cohort and cross-sectional studies.

Note: An Explanation and Elaboration article discusses each checklist item and gives methodological background and published examples of transparent reporting. The STROBE checklist is best used in conjunction with this article (freely available on the Web sites of PLoS Medicine at http://www.plosmedicine.org/, Annals of Internal Medicine at

For peer review only

http://www.annals.org/, and Epidemiology at http://www.epidem.com/). Information on the STROBE Initiative is available at www.strobe-statement.org.

For peer review only

For peer review only - http://bmjopen.bmj.com/site/about/guidelines.xhtml

BMJ Open

Association between early child development trajectories and adult cognitive function in a 50-year prospective study in Guatemala

Journal:	*BMJ Open*
Manuscript ID	bmjopen-2020-044966.R1
Article Type:	Original research
Date Submitted by the Author:	17-Mar-2021
Complete List of Authors:	Gonzalez Casanova, Ines; Indiana University Bloomington, Applied Health Science; Emory University,<br>DiGirolamo, Ann ; Georgia State University<br>Kroker-Lobos, Maria; INCAP<br>Ochaeta, Laura; INCAP<br>Ramirez-Zea, Manuel; INCAP<br>Martorell, Reynaldo; Emory University<br>Stein, Aryeh D.; Emory University,
**Primary Subject Heading**:	Mental health
Secondary Subject Heading:	Epidemiology, Paediatrics
Keywords:	EPIDEMIOLOGY, International health services < HEALTH SERVICES ADMINISTRATION & MANAGEMENT, MENTAL HEALTH, Developmental neurology & neurodisability < PAEDIATRICS, PUBLIC HEALTH

SCHOLARONE™
Manuscripts

I, the Submitting Author has the right to grant and does grant on behalf of all authors of the Work (as defined in the below author licence), an exclusive licence and/or a non-exclusive licence for contributions from authors who are: i) UK Crown employees; ii) where BMJ has agreed a CC-BY licence shall apply, and/or iii) in accordance with the terms applicable for US Federal Government officers or employees acting as part of their official duties; on a worldwide, perpetual, irrevocable, royalty-free basis to BMJ Publishing Group Ltd ("BMJ") its licensees and where the relevant Journal is co-owned by BMJ to the co-owners of the Journal, to publish the Work in this journal and any other BMJ products and to exploit all rights, as set out in our licence.

The Submitting Author accepts and understands that any supply made under these terms is made by BMJ to the Submitting Author unless you are acting as an employee on behalf of your employer or a postgraduate student of an affiliated institution which is paying any applicable article publishing charge ("APC") for Open Access articles. Where the Submitting Author wishes to make the Work available on an Open Access basis (and intends to pay the relevant APC), the terms of reuse of such Open Access shall be governed by a Creative Commons licence – details of these licences and which Creative Commons licence will apply to this Work are set out in our licence referred to above.

Other than as permitted in any relevant BMJ Author's Self Archiving Policies, I confirm this Work has not been accepted for publication elsewhere, is not being considered for publication elsewhere and does not duplicate material already published. I confirm all authors consent to publication of this Work and authorise the granting of this licence.

For peer review only

Title: Trajectories of child development predict adult cognitive function: a 50-year prospective study in Guatemala

Authors: Ines Gonzalez-Casanova[1,2], Ann DiGirolamo[3], Maria F. Kroker-Lobos[4], Laura Ochaeta[4], Manuel Ramirez-Zea[4], Reynaldo Martorell[2], Aryeh D. Stein[2]*,

1. Department of Applied Health Sciences, Indiana University Bloomington School of Public Health, IN, USA, 2. Hubert Department of Global Health, Rollins School of Public Health, Emory University, Atlanta, GA, USA, 3. Georgia Health Policy Center, Andrew Young School of Policy Studies, Georgia State University, Atlanta, Georgia, USA, 4. INCAP Research Center for the Prevention of Chronic Diseases, Institute of Nutrition of Central America and Panama, Guatemala City, Guatemala

***Corresponding Author**
Aryeh D. Stein, PhD
email: aryeh.stein@emory.edu

Article Summary

Strengths and limitations of this study

- Repeated measures of cognitive function from 6 months to 55 years of age
- Birth cohort with 50 years of follow-up from Guatemala, a transitioning country
- Longitudinal analysis using latent class growth analysis
- There was missing information from some participants
- Results are consistent with what has been reported in studies from high-income countries

Abstract

Background: in order to develop effective interventions to improve life-course cognition, it is important to understand the relationship between early childhood development and adult cognitive functioning in diverse populations. Few studies have used longitudinal data to study the development of cognition over the life course in low- and middle- income countries.
Methods: We analyzed data from the INCAP Nutrition Supplementation Trial Longitudinal Study in Guatemala. Cognition was assessed at eight different time-points between 6 months and 7 years. We derived childhood development trajectories using latent class growth analysis. We assessed predictors of the trajectories using ordinal logistic regression, and associations between childhood trajectories and adult non-verbal intelligence and literacy at age 11 to 55 years (mean ± SD =42.7 ± 6.4 years) using generalized linear models.
Results: Among 927 participants, we identified three trajectories of cognitive development (high: n=214, average: n=583, low: n=130). Participants whose mothers were taller [proportional log odds (PO)=0.03 95%CI=(0.01, 0.06)], had more years of schooling [PO=0.15 95%CI = (0.06, 0.25)], or lived in households with higher socioeconomic scores [PO = 0.19 95%CI=(0.09, 0.29)] were more likely to follow higher trajectories.
Conclusions: In this sample from Guatemala, cognitive development trajectories from 6 months through 7 years were associated with adult non-verbal intelligence and literacy. These findings provide evidence of tracking of cognition over-time in a transitioning low-income setting.

For peer review only - http://bmjopen.bmj.com/site/about/guidelines.xhtml

INTRODUCTION

In low- and middle- income (LMIC) countries, 43% of children younger than five years were considered at risk of poor development in 2010 using stunting and poverty as proxies. [1] Early childhood development and childhood intellectual functioning predict survival, lifelong health, and human capital; [2-5] hence, assuring that children worldwide reach their full cognitive developmental potential is central to the sustainable development agenda. [1]

This has led to a widespread call to implement effective interventions that improve early cognitive development.[1, 6, 7] However, the complex nature of the known predictors of early development and challenges assessing development and intelligence across time in diverse populations have complicated the targeting and evaluation of interventions to improve life-course cognitive functioning , especially in LMIC.[8, 9]

Understanding how cognitive functioning tracks across time in diverse settings is an important step towards identifying effective approaches to improve childhood development; however, information from LMIC is limited. Considering that LMIC have the greatest proportion of children at risk and are primary targets for interventions and that the first years of life are the most important period for brain growth and differentiation, it is important to address these gaps in knowledge from LMIC. Following a life-course approach and taking advantage of a long-running cohort from Guatemala, [10] the objective of this study was to assess childhood cognitive developmental trajectories during the first seven years of life, to identify predictors of these trajectories, and to estimate the associations of these trajectories with adult cognitive functioning.

For peer review only

METHODS

Longitudinal study

We used data from the INCAP Nutrition Supplementation Trial Longitudinal Study [10]. The study was originally conceived as a trial to test the hypothesis that providing a protein-energy supplement during the preschool years would improve mental development. The specific methods for the original trial have been described extensively; [10-13] briefly, four villages in Guatemala were assigned to receive either a protein-energy gruel (Atole) or a protein-free sugary drink (Fresco). The supplements were provided to all residents in the study villages in a central location twice daily from 1969 through 1977; intakes were recorded for pregnant and lactating women and children under the age of 7 years. In total, 2392 children were enrolled in the original study because they were either younger than seven years at the beginning of the trial or were born during its implementation; hence, participants in this cohort were born between 1962 and 1977 in the study villages.[14] Participants and community were not involved in the design and recruitment for the intervention. Follow-up studies have been conducted in 1988-99, 2002-04, and 2015-17. [10, 15, 16] P

Analytical Sample and Data for this Analysis

We included all participants with at least three measures of cognitive development out of the eight potentially available. This is a conservative approach as latent class growth analysis can handle some participants having as few as one measurement. [17] For the analysis testing associations with adult intelligence and literacy, we excluded participants missing information on these measurements. Information on sociodemographic predictors such as parents' years of

For peer review only

schooling, household socioeconomic status, and distance to the health center was extracted from data collected during the original supplementation study (between 1969 and 1977).

Measures

Cognitive Development Assessments

The cognitive function assessments for infants and preschool children were specifically developed for the study using items from different age-appropriate tests.[18]

Infant battery (administered at child ages 6, 15 and 24 months)-

This set of instruments was administered within a two-week window of infants turning 6, 15, and 24 months. It was developed as a compendium of relevant items from validated infant scales including the Bayley Scales of Infant Development [19], the Gesell and Amatruda's Developmental Diagnosis [20], the Cattell Infant's Intelligence Scales [21], and Merrill-Palmer Standards of Physical and Mental Growth [22]. The selected items were translated into Spanish and adapted for the Guatemalan context by trained psychologists and psychiatrists. The Composite Infant Score (CIS) summarizes 44 items derived from the previously mentioned scales.

Preschool battery (administered at child ages 3, 4, 5, 6 and 7 years) -

A set of 22 tests was applied within a month of the participants' fifth, sixth and seventh birthday, and a shorter set of 10 tests was applied within a month of children's third and fourth birthdays. The preschool battery measured various dimensions of cognition (e.g. verbal ability, general knowledge, quantitative skills and memory). These scales have previously been analyzed by

For peer review only

factor analysis, resulting in a general and a memory factor, which were deemed reliable and valid predictors of later development.[23]

Raven's Progressive Matrices (RPM) –

The RPM was applied during the 1988-1989, 2004-2005, and 2015-2017 data collection waves, when participants were between 11-27, 22-43, and 37-55 years old, respectively. The RPM is a non-verbal test of intellectual functioning and abstract reasoning that includes a series of pattern-matching exercises that get progressively more complex.[24] The RPM traditionally consists of five sets of 12 items each, however only the first three sets were applied, because in our pilot work we found that few participants could process further than that. [14] The score was calculated by summing the number of correct answers. The RPM has good test-retest reliability (intra-class correlation coefficient, 0.87) and internal consistency, as well as construct validity in this Guatemalan population.[14]

Adult literacy and reading comprehension

InterAmerican Reading Series (Serie Interamericana) –

This test was administered in 2004-2005 and 2015-2017. To complete this test, those who were unable to read a test sentence from the local paper or had not completed primary school, received the pre-screening test questions where scores could range between zero and five. Those who passed the screening test (by answering the five questions correctly) or had at least a secondary school education started with a score of 5 which was then added to the InterAmerican reading and writing series composite score. This approach has been found to have adequate test-retest reliability and internal consistency in this population [25].

Statistical Analysis

For peer review only - http://bmjopen.bmj.com/site/about/guidelines.xhtml

Summarizing child cognitive data into trajectories

In order to make the scores comparable over time, we first converted the individual scores for mental development on the CIS at 6, 15 and 24 months, and the preschool series composite score at 3, 4, 5, 6, and 7 years into z-scores relative to the same population and age. We assessed Spearman pairwise correlations between all the available childhood cognitive development measurements during the first seven years and RPM measurements stratified by age. We then used LCGA [17] to cluster participants into developmental trajectories during the first seven years based on the age-adjusted ranked scores. LGCA classifies individuals based on their cognitive development at different ages into classes or trajectories with distinct intercepts and slopes, thereby capturing the heterogeneity within a sample. Because of the way the original study was designed, in order to account for potentially systematically missing information by birth year (supplemental table 1), a sensitivity analysis was conducted by including children with at least one CIS and at least one preschool measurement. All LCGA were adjusted for sex and for maternal ID (to account for siblings in the sample). The LCGA analysis was performed using M-Plus (Version 7.0, Los Angeles, CA).

Assessing predictors of child cognitive development trajectories:

We assessed if family-level variables predicted the trajectories using ordinal logistic regression. Models were developed based on theory and available data on the household sociodemographic determinants of child development, which included SES score, parents' education and height, family size (number of people living in the same household), and distance from the supplementation center. All models were adjusted for type of supplement (operationalized as village of residence) and age at exposure to the supplement (first 1000 days vs. not within the first 1000 days). Multiple imputation in SAS 9.4 was conducted to account for item-specific

For peer review only - http://bmjopen.bmj.com/site/about/guidelines.xhtml

missing values in the predictors. We used PROC MI to generate twenty imputed datasets using fully conditional specification. Ordinal logistic regression was then conducted for each of the twenty imputed datasets and the estimates were pooled using PROC MIANALYZE.

Testing associations between child development measurements, trajectories and adult outcomes

We used the last available RPM measure (mean age ± SD = 42.7 ± 6.4). For the literacy score, we used the last score recorded. We used ANOVA to assess mean RPM and literacy score differences by trajectory and by 7-year tertiles as comparison.

Data management and analysis were conducted using SAS 9.4 (SAS Institute Inc., Cary, NC, USA) unless otherwise noted.

RESULTS

The sample for the trajectory analysis and early predictors included 927 participants with at least three measurements of cognitive development during childhood (Supplemental Table 1). Sample sizes for testing associations between trajectory membership and adult cognitive functioning varied; in total, 823 participants could be classified into childhood trajectories and had at least one RPM measurement (Figure 1).

[Figure 1- Sample flowchart for cognitive development trajectory analysis in the INCAP Nutrition Supplementation Trial Longitudinal Study in Guatemala]

In the LCGA, based on model fit indexes, the sample membership for each trajectory and interpretability of the resulting classes, we identified three trajectories which we labeled low, average, and high (Figure 2). A sensitivity analysis including only children with at least one infant and one preschool measurement (n=577) also resulted in three similar trajectories.

For peer review only - http://bmjopen.bmj.com/site/about/guidelines.xhtml

Differences among the three trajectories were apparent already at 6 months and became larger as the children grew older.

[Figure 2: Cognitive development trajectories from 6 months to 7 years (84 months) of age (n=927)]

The weakest Spearman correlations with the adult RPM assessments were with the infant battery at 6 and 15 months and with the preschool battery at 3 years, followed by the infant battery at 2 years and the preschool battery at 4 years. All the correlations between the 5-, 6-, and 7-year measurements, the trajectories, and the adult RPM were in the moderate range. The strongest correlations between childhood assessments and the RPM were with the preschool battery at 6 years, which ranged between 0.40 (at 11-20 and 21-36 years) and 0.43 (at 37-55 years). The 7-year preschool battery had the strongest correlation with the RPM at ages 11-20 years; the trajectories had the weakest correlation with measurements at 11-20 years (0.30) but stronger correlations with the RPM at 21-36 years (0.35) and 37-55 years (0.38). The RPM at 11-20 years had a correlation of 0.54 with the RPM at 37 to 55 years (Table 1).

Participants in the high trajectory belonged to households that, on average, had higher socioeconomic status, had mothers and fathers with more years of schooling, and had taller mothers, relative to those in the average trajectory. Conversely, the low trajectory had, on average, lower household socioeconomic status, their parents had fewer years of schooling and their mothers were shorter than those in the average trajectory (Table 2).

In proportional odds cumulative logit models, maternal and paternal education and height, and SES were positively associated with the trajectories, while family size was inversely associated with the trajectories (Table 3). After adjusting for all other covariates, the positive association

For peer review only - http://bmjopen.bmj.com/site/about/guidelines.xhtml

with maternal schooling, maternal height and household SES remained, as did the inverse association with family size.

Child development trajectories were associated with adult RPM and literacy scores (Table 4). The mean and standard deviation scores for the latest RPM were 16.0 and 6.3, respectively; on average, participants in the low childhood cognitive development trajectory scored 5.6 fewer points on the RPM compared to those in the high trajectory (a difference of 0.93 standard deviations of the mean). The mean and standard deviation scores for the latest literacy scores were 49.8 and 28.6, respectively. The mean difference between the low and high trajectory for literacy scores was 29.6 (which represents 1.03 standard deviations of the mean). The 7-year cognitive development score tertiles were also associated with RPM and literacy scores in adulthood. Those in the highest 7-year tertile scored on average 4.9 points higher on the RPM and 25.8 points higher on the literacy test compared to those in the lowest tertile.

DISCUSSION

The primary aim of this study was to use a life-course approach to assess child development longitudinally in a longstanding cohort from Guatemala, which has 50 years of follow-up, and, in line with this approach, to identify predictors of early development and correlations with adult non-verbal intelligence and literacy. To our knowledge, this is the first study to assess life-course measures of cognition from early life through adulthood in a LMIC using a developmental trajectory approach, assessing relationships over time.

We identified three trajectories of childhood cognitive development through age seven years that diverge early and then remain parallel through the early school years. Consistent with this finding, early factors such as household SES and family size at birth, as well as maternal years of

For peer review only - http://bmjopen.bmj.com/site/about/guidelines.xhtml

schooling and height were associated with trajectory membership, independently of other sociodemographic variables. These findings support early life as a critical period and highlight the importance of holistic interventions that address the complex interplay between sociodemographic factors, which ultimately can determine lifelong health advantages or vulnerabilities [26]. They also highlight the importance of investing in maternal education and nutrition within a context of a transitioning society to break the inter-generational transmission of inequality.

Results from this analysis, besides supporting early life as a critical period for the development of life-course cognition, also show a cumulative process where single time-point measurements at older ages (5-7 years) have stronger correlations with adult outcomes when compared to measurements conducted during infancy or early childhood (<4 years). The trajectory approach also provided more weight to the information from measurements conducted later in childhood. Taken together, results from this study highlight the importance of child cognitive development as a potential pathway linking early exposures and adult outcomes. This is important for the design and assessment of interventions and programs aiming to improve life-course development, health, human capital, as well as economic development.

We found mid-size correlations of 0.38 between cognitive development trajectories through seven years and non-verbal intelligence measured after 37 years of age. The trajectories created using repeated measurements of cognitive development by different instruments performed similarly to a single time point measurement at age 7 years of cognitive functioning in predicting adult intelligence and literacy in this sample from Guatemala. The use of trajectories has the added advantage of maximizing the sample size, which can decrease bias. The stability of the trajectories decreases the need to conduct multiple tests that increase the risk of chance findings.

For peer review only - http://bmjopen.bmj.com/site/about/guidelines.xhtml

Similarly, there is no consensus on the best age to assess the impact of interventions on development or to predict later cognitive outcomes as this might vary by setting and population;[1, 27, 28] trajectories allow utilizing all available information rather than relying on a single cross-sectional measurement. Another advantage of using LCGA was that we were able to interpret the trajectories and make qualitative inferences about the timing and processes of early cognition within sub-categories of this sample. For example, as mentioned before, the early separation of the trajectories highlights the importance of timing interventions during the prenatal period or early life.

The analysis of the correlation among the trajectories, individual childhood measurements, and adult outcomes also provided insights into the predictive value of individual time-measurements at different ages, which would not be evident with single measurements. In this instance, we showed higher correlations of individual child development measurements after 5 years with both the trajectories and adult non-verbal intelligence, when compared to earlier measurements. These results could be reflective of methodological challenges assessing development in younger children, [11, 29] as well as of cumulative processes and pathways that eventually lead to adult cognition.

Missing data is a limitation of this study. Less than half of the participants of the original study had three or more measurements of cognitive development and, due to the study design, most participants have missing data either at younger or older childhood measurements. However, to account for this, we conducted a sensitivity analysis including only those participants with at least one measurement during infancy and at least one measurement during the preschool period and found similar trajectories. Another limitation with the study design was the large age-span of the cohort participants (i.e., 11 years), which forced us to include large age groups for adult

For peer review only - http://bmjopen.bmj.com/site/about/guidelines.xhtml

measurements and complicated the study of specific periods during adulthood. A limitation of the LCGA approach is that the researcher selects the number of classes. We used model fit statistics and class size to select the number of classes but the process is empirical and not necessarily exempt from bias. Finally, there are additional factors that could have contributed to child cognitive development that were not measured in this study; for example, information on parents' intelligence or early childhood care and stimulation, among others factors, were not available.

Global estimates of the number of children not meeting their full developmental potential are currently based on poverty and stunting.[1, 30] Even though these two factors are associated with early childhood development, the use of proxies to assess cognition is limiting [9], and addressing this limitation is important in order to improve our ability to study the determinants of life-course cognition.[9] Results from this study contribute to further our understanding of cognitive functioning across the lifespan in LMIC. This cognitive development trajectory approach sets the basis for the study of life-course predictors of intelligence, which in turn can improve the design and targeting of interventions in diverse populations.

Funding: This study was funded by the Bill and Melinda Gates Foundation OPP1164115. The funder played no role in the design, implementation or interpretation of this study.

For peer review only - http://bmjopen.bmj.com/site/about/guidelines.xhtml

Table 1: Spearman pairwise correlations between cognitive development tests from 6 months through adulthood (n=1484 with at least 2 measurements of intelligence)

	r(n)[a]	Infant Battery			Preschool Battery						Raven Progressive Matrices		
		**6 mo**	**15 mo**	**24 mo**	**3 y**	**4 y**	**5 y**	**6 y**	**7 y**	**Trajectories**	**11-20 y**	**21-36 y**	**37-55 y**
**INFANT**	**6 mo**	*1.00* (397)	0.28 (312)	0.14 (293)	0.02 (344)	0.03 (342)	0.11 (277)	0.03 (180)	-0.11 (88)	0.14 (361)	0.08 (266)	0.11 (289)	0.08 (201)
	**15 mo**		*1.00* (469)	0.28 (312)	0.09 (425)	0.02 (425)	0.17 (332)	0.05 (222)	0.09 (129)	0.21 (447)	0.04 (325)	0.14 (345)	0.11 (236)
	**24 mo**			*1.00* (466)	0.36 (431)	0.09 (426)	0.16 (361)	0.28 (256)	0.25 (165)	0.39 (452)	0.11 (296)	0.21 (352)	0.18 (238)
**PRESCHOOL**	**3 y**				*1.00* (736)	0.29 (692)	0.29 (570)	0.26 (444)	0.24 (329)	0.45 (691)	0.03 (398)	0.09 (567)	0.03 (388)
	**4 y**					*1.00* (860)	0.41 (696)	0.33 (567)	0.31 (443)	0.53 (806)	0.13 (414)	0.21 (656)	0.23 (450)
	**5 y**						*1.00* (833)	0.60 (664)	0.54 (533)	0.71 (793)	0.35 (328)	0.34 (596)	0.32 (443)
	**6 y**							*1.00* (791)	0.66 (632)	0.77 (663)	0.43 (231)	0.40 (502)	0.40 (440)
	**7 y**								*1.00* (739)	0.76 (540)	0.45 (140)	0.34 (409)	0.35 (422)
	**Trajectories**[b]									1.00 (927)	0.30 (409)	0.36 (667)	0.38 (495)
**RAVEN**	**11-20y**										*1.00* (1297)	0.57 (753)	0.54 (523)
	**21-36y**											*1.00* (1452)	0.96 (855)
	**37-55y**												*1.00* (1163)

[a]Values are Spearman correlations between child z-scores and adult raw raven scores.
[b]The trajectories were coded low=0, average=1 and high=2 for this analysis.

For peer review only - http://bmjopen.bmj.com/site/about/guidelines.xhtml

Table 2: Sociodemographic and childhood characteristics by cognitive development trajectory

	**Low (130)**	**Average (583)**	**High (214)[a]**	**p-value**
Community-level predictor				
Large village (%)	56.1	55.3	55.0	0.97
Household-level predictors at enrollment				
Distance from supplementation center (Km)	2.7 ± 0.1	2.5 ± 0.1	2.4 ± 0.1	0.26
SES Score	-0.5 ± 0.1	-0.2 ± 0.1	0.2 ± 0.1	<0.001
Maternal schooling (years)	1.0 ± 0.1	1.2 ± 0.1	1.8 ± 1.1	<0.001
Paternal schooling (years)	1.3 ± 0.2	1.5 ± 0.1	2.0 ± 0.1	<0.001
Maternal height (cm)	148.0 ± 0.5	148.9 ± 0.2	150.0 ± 0.4	<0.001
Paternal height (cm)	160.1 ± 0.6	160.6 ± 0.3	161.6 ± 0.6	0.12
Family size	6.8 ± 0.2	6.7 ± 0.1	6.5 ± 0.2	0.34
Individual-level predictors				
Male (%)	52.6	51.6	54.2	0.83
Height 36 mo (cm)	83.5 ± 0.4	85.5 ± 0.2	86.7 ± 0.3	<0.001
Weight 36 mo (kg)	11.3 ± 0.2	11.8 ± 0.1	12.0 ±0.1	<0.001

[a]Trajectory analysis was adjusted by sex and age at measurement. Differences among groups were assessed using chi-square test for categorical variables and ANOVA for continuous variables.

For peer review only

For peer review only - http://bmjopen.bmj.com/site/about/guidelines.xhtml

Table 3: Proportional odds logistic regression of sociodemographic predictors and cognitive development trajectories (n=927)[a]

	Unadjusted		Adjusted[b]	
	Estimate[a]	95% CI	Estimate[a]	95% CI
SES Score	0.22	(0.13, 0.32)	0.19	(0.09, 0.29)
Family size (# of people)	-0.06	(-0.13, 0.00)	-0.08	(-0.16, -0.01)
Distance from supplementation center (Km)	-0.09	(-0.21, 0.03)	-0.03	(-0.16, 0.09)
Parents' schooling (years)				
Maternal	0.20	(0.12, 0.29)	0.15	(0.06, 0.25)
Paternal	0.12	(0.05, 0.18)	0.07	(0.00, 0.15)
Parents' height (cm)				
Maternal	0.04	(0.02, 0.07)	0.03	(0.01, 0.06)
Paternal	0.03	(0.00, 0.05)	0.01	(-0.02, 0.04)

[a] Estimates represent the proportional log odds of following a higher trajectory per unit increase in the predictor. Multiple imputation was used for item-specific missing values in the covariates. Trajectories were coded as low=0, average=1, high=2;

[b] Adjusted for all other covariates and for supplementation with Atole in the first 1000 days (sex and age at measurement were adjusted during the LCGA and thus were not included in this model).

For peer review only - http://bmjopen.bmj.com/site/about/guidelines.xhtml

Table 4: Associations between cognitive development trajectories, preschool series tertiles at 7 years, and adult literacy and non-verbal intelligence

	Trajectory [d]				7-years scores [e]			
	n	Low	Average	High	n	Low	Medium	High
Last available literacy score [a, c]	796	33.8 ± 2.6	48.2 ± 1.2	63.4 ± 2.0	604	33.6 ± 1.9	45.9 ± 1.9	59.4 ± 1.9
Last available non-verbal intelligence score[c]	823	13.8 ± 0.6	15.4 ± 0.3	19.4 ± 0.5	604	13.1 ± 0.4	14.9 ± 0.4	18.0 ± 0.4

[a]Measured by the InterAmerican Series;

[b]Measured by the Raven Progressive Matrices test;

[c]Mean ± standard deviation age at last measurement was 42.7 ± 6.4 years;

[d] Values are means and standard errors, mean differences by trajectory were assessed using ANOVA (all p-values <0.001);

[e]Values are means and standard errors by Preschool series 7-year score tertiles, mean differences by tertile were assessed using ANOVA (all p-values <0.001).

For peer review only - http://bmjopen.bmj.com/site/about/guidelines.xhtml

STATEMENTS

A. Contributorship statement

All authors contributed substantially to the conception of the work, acquisition, analysis and/or interpretation of the data as required by BMJ authorship requirements. IGC drafted the original manuscript and all authors reviewed it critically for intellectual content. All authors agree to be accountable for all aspects of the work reported in this manuscript.

B. Competing Interests

Authors declare no competing interests

C. Funding

This study was funded by the Bill and Melinda Gates Foundation (#OPP1164115). The funder was not involved in the design or interpretation of the results of this analysis.

D. Data Sharing statement

Data are available from the corresponding author upon written request.

REFERENCES

1 Black MM, Walker SP, Fernald LCH*, et al.* Early childhood development coming of age: science through the life course. *The Lancet* 2017;**389**:77-90.

2 Wraw C, Deary IJ, Gale CR*, et al.* Intelligence in youth and health at age 50. *Intelligence* 2015;**53**:23-32.

3 Jokela M, Elovainio M, Singh-Manoux A*, et al.* IQ, socioeconomic status, and early death: The US National Longitudinal Survey of Youth. *Psychosomatic medicine* 2009;**71**:322-8.

4 Calvin CM, Deary IJ, Fenton C*, et al.* Intelligence in youth and all-cause-mortality: systematic review with meta-analysis. *International journal of epidemiology* 2011;**40**:626-44.

5 McHutchison CA, Backhouse EV, Cvoro V*, et al.* Education, Socioeconomic Status, and Intelligence in Childhood and Stroke Risk in Later Life: A Meta-analysis. *Epidemiology (Cambridge, Mass)* 2017;**28**:608-18.

6 Richards M, Deary IJ. A life course approach to cognitive reserve: a model for cognitive aging and development? *Annals of neurology* 2005;**58**:617-22.

7 Kuh D, Network* tNDoAP. A Life Course Approach to Healthy Aging, Frailty, and Capability. *The Journals of Gerontology: Series A* 2007;**62**:717-21.

8 Sabanathan S, Wills B, Gladstone M. Child development assessment tools in low-income and middle-income countries: how can we use them more appropriately? *Archives of disease in childhood* 2015;**100**:482-8.

9 Richter LM, Black M, Britto P*, et al.* Early childhood development: an imperative for action and measurement at scale. *BMJ global health* 2019;**4**:e001302.

10 Stein AD, Melgar P, Hoddinott J*, et al.* Cohort Profile: the Institute of Nutrition of Central America and Panama (INCAP) Nutrition Trial Cohort Study. *International journal of epidemiology* 2008;**37**:716-20.

For peer review only - http://bmjopen.bmj.com/site/about/guidelines.xhtml

11 Hack M, Taylor HG, Drotar D, *et al.* Poor predictive validity of the Bayley Scales of Infant Development for cognitive function of extremely low birth weight children at school age. *Pediatrics* 2005;**116**:333-41.
12 Martorell R, Habicht JP, Rivera JA. History and design of the INCAP longitudinal study (1969-77) and its follow-up (1988-89). *J Nutr* 1995;**125**:1027s-41s.
13 Ramirez-Zea M, Mazariegos M. INCAP Longitudinal Study: 50 Years of History and Legacy. *Food and Nutrition Bulletin* 2020;**41**:S5-S7.
14 Pollitt E, Gorman KS, Engle PL, *et al.* Early Supplementary Feeding and Cognition: Effects over Two Decades. *Monographs of the Society for Research in Child Development* 1993;**58**:i-118.
15 Ford ND, Martorell R, Mehta NK, *et al.* Life-Course Body Mass Index Trajectories Are Predicted by Childhood Socioeconomic Status but Not Exposure to Improved Nutrition during the First 1000 Days after Conception in Guatemalan Adults. *The Journal of nutrition* 2016;**146**:2368-74.
16 Ford ND, Behrman JR, Hoddinott JF, *et al.* Exposure to improved nutrition from conception to age 2 years and adult cardiometabolic disease risk: a modelling study. *The Lancet Global Health* 2018;**6**:e875-e84.
17 Jung T, Wickrama KAS. An Introduction to Latent Class Growth Analysis and Growth Mixture Modeling. *Social and Personality Psychology Compass* 2008;**2**:302-17.
18 Engle PL, Fernandez PD. INCAP studies of malnutrition and cognitive behavior. *Food Nutr Bull* 2010;**31**:83-94.
19 Bayley N. *Bayley scales of infant development*. New York: The Psychological Corporation 1969.
20 Knobloch H, Stevens FM, Malone AF, *et al. Manual of developmental diagnosis : the administration and interpretation of the revised Gesell and Amatruda developmental and neurologic examination*. Hagerstown, Md.: Harper & Row 1980.
21 Cavanaugh MC, Cohen I, Dunphy D, *et al.* Prediction from the Cattell infant intelligence scale. *J Consult Psychol* 1957;**21**:33-7.
22 Merrill-Palmer Institute., Wilson CA. *The Merrill-Palmer standards of physical and mental growth*. Detroit 1930.
23 Pollitt E, Triana N. Stability, Predictive Validity, and Sensitivity of Mental and Motor Development Scales and Pre-School Cognitive Tests among Low-Income Children in Developing Countries. *Food and Nutrition Bulletin* 1999;**20**:45-52.
24 Raven J, Raven J. Raven Progressive Matrices. *Handbook of nonverbal assessment*. New York, NY, US: Kluwer Academic/Plenum Publishers 2003:223-37.
25 Stein AD, Wang M, DiGirolamo A, *et al.* Nutritional supplementation in early childhood, schooling, and intellectual functioning in adulthood: a prospective study in Guatemala. *Arch Pediatr Adolesc Med* 2008;**162**:612-8.
26 Maggi S, Irwin LJ, Siddiqi A, *et al.* The social determinants of early child development: An overview. *Journal of Paediatrics and Child Health* 2010;**46**:627-35.
27 Grantham-McGregor S, Cheung YB, Cueto S, *et al.* Developmental potential in the first 5 years for children in developing countries. *Lancet* 2007;**369**:60-70.
28 Prado EL, Dewey KG. Nutrition and brain development in early life. *Nutrition Reviews* 2014;**72**:267-84.
29 Aslin RN, Fiser J. Methodological challenges for understanding cognitive development in infants. *Trends in Cognitive Sciences* 2005;**9**:92-8.
30 Lu C, Black MM, Richter LM. Risk of poor development in young children in low-income and middle-income countries: an estimation and analysis at the global, regional, and country level. *The Lancet Global Health* 2016;**4**:e916-e22.

For peer review only - http://bmjopen.bmj.com/site/about/guidelines.xhtml

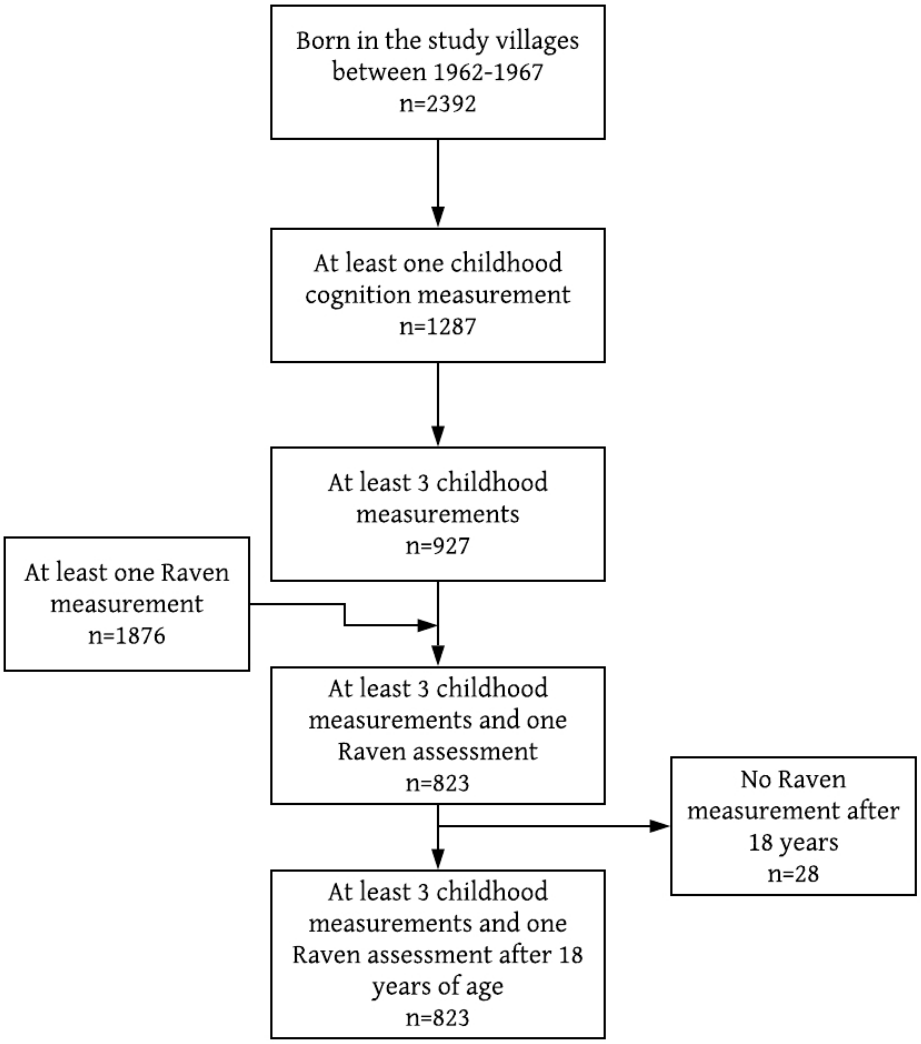

Figure 1- Sample flowchart for cognitive development trajectory analysis in the INCAP Nutrition Supplementation Trial Longitudinal Study in Guatemala

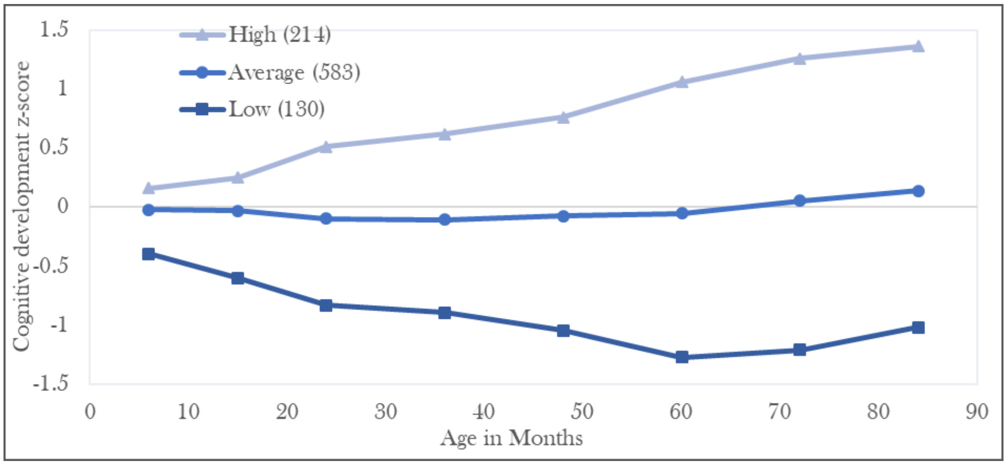

Figure 2: Cognitive development trajectories from 6 months to 7 years (84 months) of age (n=927)

Supplemental Table 1: Sample size of childhood cognitive development and adult Raven Progressive Matrices assessments by birth year in the INCAP Nutrition Supplementation Trial Longitudinal Study (n=2392)

Year of birth	Infant development scales			Preschool Series					≥ 3 child assessments	≥ 1 child assessments	≥ 1 adult assessments	≥ 1 adult and >1 child assessments
	6 months	15 months	24 months	3 years	4 years	5 years	6 years	7 years				
1962	0	0	0	0	0	0	0	47	0	47	60	39
1963	0	0	0	0	0	0	66	86	0	89	80	69
1964	0	0	0	0	0	53	73	78	47	83	63	54
1965	0	0	0	0	63	91	96	97	88	105	97	86
1966	0	0	0	66	98	100	104	101	99	116	113	99
1967	0	1	34	78	92	94	92	96	92	110	112	94
1968	12	44	57	93	95	100	95	94	97	117	114	96
1969	76	84	84	113	118	120	122	122	125	145	146	126
1970	97	95	92	112	113	110	117	18	118	141	143	124
1971	113	116	106	125	128	131	26	0	130	156	146	126
1972	99	114	93	124	129	34	0	0	117	152	168	136
1973	0	15	0	25	24	0	0	0	14	26	133	23
1974	0	0	0	0	0	0	0	0	0	0	148	0
1975	0	0	0	0	0	0	0	0	0	0	133	0
1976	0	0	0	0	0	0	0	0	0	0	135	0
1977	0	0	0	0	0	0	0	0	**0**	0	84	0
**Total**	**397**	**469**	**466**	**736**	**860**	**833**	**791**	**739**	**927**	**1287**	**1875**	**1072**

For peer review only

STROBE Statement—checklist of items that should be included in reports of observational studies

	Item No	Recommendation	Page No
**Title and abstract**	1	(*a*) Indicate the study's design with a commonly used term in the title or the abstract	1
		(*b*) Provide in the abstract an informative and balanced summary of what was done and what was found	1
**Introduction**			
Background/rationale	2	Explain the scientific background and rationale for the investigation being reported	2
Objectives	3	State specific objectives, including any prespecified hypotheses	2
**Methods**			
Study design	4	Present key elements of study design early in the paper	3
Setting	5	Describe the setting, locations, and relevant dates, including periods of recruitment, exposure, follow-up, and data collection	3
Participants	6	(*a*) *Cohort study*—Give the eligibility criteria, and the sources and methods of selection of participants. Describe methods of follow-up<br>*Case-control study*—Give the eligibility criteria, and the sources and methods of case ascertainment and control selection. Give the rationale for the choice of cases and controls<br>*Cross-sectional study*—Give the eligibility criteria, and the sources and methods of selection of participants	3
		(*b*) *Cohort study*—For matched studies, give matching criteria and number of exposed and unexposed<br>*Case-control study*—For matched studies, give matching criteria and the number of controls per case	NA
Variables	7	Clearly define all outcomes, exposures, predictors, potential confounders, and effect modifiers. Give diagnostic criteria, if applicable	4,5
Data sources/ measurement	8*	For each variable of interest, give sources of data and details of methods of assessment (measurement). Describe comparability of assessment methods if there is more than one group	*4,5*
Bias	9	Describe any efforts to address potential sources of bias	6
Study size	10	Explain how the study size was arrived at	4
Quantitative variables	11	Explain how quantitative variables were handled in the analyses. If applicable, describe which groupings were chosen and why	5,6
Statistical methods	12	(*a*) Describe all statistical methods, including those used to control for confounding	5,6
		(*b*) Describe any methods used to examine subgroups and interactions	5,6
		(*c*) Explain how missing data were addressed	5,6
		(*d*) *Cohort study*—If applicable, explain how loss to follow-up was addressed<br>*Case-control study*—If applicable, explain how matching of cases and controls was addressed<br>*Cross-sectional study*—If applicable, describe analytical methods taking account of sampling strategy	NA
		(*e*) Describe any sensitivity analyses	6

Continued on next page

For peer review only - http://bmjopen.bmj.com/site/about/guidelines.xhtml

**Results**			
Participants	13*	(a) Report numbers of individuals at each stage of study—eg numbers potentially eligible, examined for eligibility, confirmed eligible, included in the study, completing follow-up, and analysed	7, Figure 1, Supplemental table 1
		(b) Give reasons for non-participation at each stage	Figure 1
		(c) Consider use of a flow diagram	Figure 1
Descriptive data	14*	(a) Give characteristics of study participants (eg demographic, clinical, social) and information on exposures and potential confounders	7, Table 2
		(b) Indicate number of participants with missing data for each variable of interest	Table 1
		(c) *Cohort study*—Summarise follow-up time (eg, average and total amount)	NA
Outcome data	15*	*Cohort study*—Report numbers of outcome events or summary measures over time	*NA*
		Case-control study—Report numbers in each exposure category, or summary measures of exposure	*NA*
		Cross-sectional study—Report numbers of outcome events or summary measures	*NA*
Main results	16	(*a*) Give unadjusted estimates and, if applicable, confounder-adjusted estimates and their precision (eg, 95% confidence interval). Make clear which confounders were adjusted for and why they were included	8, Table 2, Table 3
		(*b*) Report category boundaries when continuous variables were categorized	NA
		(*c*) If relevant, consider translating estimates of relative risk into absolute risk for a meaningful time period	NA
Other analyses	17	Report other analyses done—eg analyses of subgroups and interactions, and sensitivity analyses	7,8
**Discussion**			
Key results	18	Summarise key results with reference to study objectives	9
Limitations	19	Discuss limitations of the study, taking into account sources of potential bias or imprecision. Discuss both direction and magnitude of any potential bias	11
Interpretation	20	Give a cautious overall interpretation of results considering objectives, limitations, multiplicity of analyses, results from similar studies, and other relevant evidence	9-11
Generalisability	21	Discuss the generalisability (external validity) of the study results	12
**Other information**			
Funding	22	Give the source of funding and the role of the funders for the present study and, if applicable, for the original study on which the present article is based	12

*Give information separately for cases and controls in case-control studies and, if applicable, for exposed and unexposed groups in cohort and cross-sectional studies.

Note: An Explanation and Elaboration article discusses each checklist item and gives methodological background and published examples of transparent reporting. The STROBE checklist is best used in conjunction with this article (freely available on the Web sites of PLoS Medicine at http://www.plosmedicine.org/, Annals of Internal Medicine at

For peer review only - http://bmjopen.bmj.com/site/about/guidelines.xhtml

http://www.annals.org/, and Epidemiology at http://www.epidem.com/). Information on the STROBE Initiative is available at www.strobe-statement.org.

For peer review only

For peer review only - http://bmjopen.bmj.com/site/about/guidelines.xhtml

BMJ Open

Association between early child development trajectories and adult cognitive function in a 50-year prospective study in Guatemala

Journal:	*BMJ Open*
Manuscript ID	bmjopen-2020-044966.R2
Article Type:	Original research
Date Submitted by the Author:	13-May-2021
Complete List of Authors:	Gonzalez Casanova, Ines; Indiana University Bloomington, Applied Health Science; Emory University,<br>DiGirolamo, Ann ; Georgia State University<br>Kroker-Lobos, Maria; INCAP<br>Ochaeta, Laura; INCAP<br>Ramirez-Zea, Manuel; INCAP<br>Martorell, Reynaldo; Emory University<br>Stein, Aryeh D.; Emory University,
**Primary Subject Heading**:	Mental health
Secondary Subject Heading:	Epidemiology, Paediatrics
Keywords:	EPIDEMIOLOGY, International health services < HEALTH SERVICES ADMINISTRATION & MANAGEMENT, MENTAL HEALTH, Developmental neurology & neurodisability < PAEDIATRICS, PUBLIC HEALTH

SCHOLARONE™
Manuscripts

I, the Submitting Author has the right to grant and does grant on behalf of all authors of the Work (as defined in the below author licence), an exclusive licence and/or a non-exclusive licence for contributions from authors who are: i) UK Crown employees; ii) where BMJ has agreed a CC-BY licence shall apply, and/or iii) in accordance with the terms applicable for US Federal Government officers or employees acting as part of their official duties; on a worldwide, perpetual, irrevocable, royalty-free basis to BMJ Publishing Group Ltd ("BMJ") its licensees and where the relevant Journal is co-owned by BMJ to the co-owners of the Journal, to publish the Work in this journal and any other BMJ products and to exploit all rights, as set out in our licence.

The Submitting Author accepts and understands that any supply made under these terms is made by BMJ to the Submitting Author unless you are acting as an employee on behalf of your employer or a postgraduate student of an affiliated institution which is paying any applicable article publishing charge ("APC") for Open Access articles. Where the Submitting Author wishes to make the Work available on an Open Access basis (and intends to pay the relevant APC), the terms of reuse of such Open Access shall be governed by a Creative Commons licence – details of these licences and which Creative Commons licence will apply to this Work are set out in our licence referred to above.

Other than as permitted in any relevant BMJ Author's Self Archiving Policies, I confirm this Work has not been accepted for publication elsewhere, is not being considered for publication elsewhere and does not duplicate material already published. I confirm all authors consent to publication of this Work and authorise the granting of this licence.

For peer review only

Title: Association between early child development trajectories and adult cognitive function in a 50-year prospective study in Guatemala

Authors: Ines Gonzalez-Casanova[1,2], Ann DiGirolamo[3], Maria F. Kroker-Lobos[4], Laura Ochaeta[4], Manuel Ramirez-Zea[4], Reynaldo Martorell[2], Aryeh D. Stein[2]*,

[1.] Department of Applied Health Sciences, Indiana University Bloomington School of Public Health, IN, USA, 2. Hubert Department of Global Health, Rollins School of Public Health, Emory University, Atlanta, GA, USA, 3. Georgia Health Policy Center, Andrew Young School of Policy Studies, Georgia State University, Atlanta, Georgia, USA, 4. INCAP Research Center for the Prevention of Chronic Diseases, Institute of Nutrition of Central America and Panama, Guatemala City, Guatemala

***Corresponding Author**
Aryeh D. Stein, PhD
email: aryeh.stein@emory.edu

Abstract
Objectives: Few studies have used longitudinal data to study the development of cognition over the life course in low- and middle- income countries. The objectives of this study were to assess predictors of cognitive development trajectories from 6 months through 7 years, and if these trajectories predicted adult cognitive function in a birth cohort from Guatemala.
Design: We analyzed data from the INCAP Nutrition Supplementation Trial Longitudinal Study in Guatemala. Cognition was assessed at eight different time-points between 6 months and 7 years. We derived childhood development trajectories using latent class growth analysis. We assessed predictors of the trajectories using ordinal logistic regression, and associations between childhood trajectories and adult non-verbal intelligence and literacy at age 18 to 52 years (mean ± SD =42.7 ± 6.4 years) using mixed models.
Setting: The study was conducted in four Guatemalan villages.
Participants: The study included 927 participants from Guatemala with repeated measurements of cognitive function during the first 7 years of life.
Results: We identified three trajectories of cognitive development (high: n=214, average: n=583, low: n=130). Participants whose mothers were taller [proportional log odds (PO)=0.03 95%CI=(0.01, 0.06)], had more years of schooling [PO=0.15 95%CI = (0.06, 0.25)], or lived in households with higher socioeconomic scores [PO = 0.19 95%CI=(0.09, 0.29)] were more likely to follow higher trajectories. Childhood trajectories predicted adult non-verbal intelligence (high=18.4 ± 0.3, average=14.6 ± 0.53, low=11.4 ± 0.9) and literacy (high=18.4 ± 0.3, average=43.6 ± 2.1, low=33.9 ± 2.6) scores.
Conclusions: In this sample from Guatemala, cognitive development trajectories from 6 months through 7 years were associated with adult non-verbal intelligence and literacy. These findings provide evidence of tracking of cognition over-time in a transitioning low-income setting.

Strengths and limitations of this study

- Repeated measures of cognitive function from 6 months to 55 years of age

For peer review only - http://bmjopen.bmj.com/site/about/guidelines.xhtml

- Birth cohort with 50 years of follow-up from Guatemala, a transitioning country
- Longitudinal analysis using latent class growth analysis
- There was missing information from some participants
- Results are consistent with what has been reported in studies from high-income countries

INTRODUCTION

In low- and middle- income (LMIC) countries, 43% of children younger than five years were considered at risk of poor development in 2010 using stunting and poverty as proxies. [1] Early childhood development and childhood intellectual functioning predict survival, lifelong health, and human capital; [2-5] hence, assuring that children worldwide reach their full cognitive developmental potential is central to the sustainable development agenda. [1]

This has led to a widespread call to implement effective interventions that improve early cognitive development.[1, 6, 7] However, the complex nature of the known predictors of early development and challenges assessing development and intelligence across time in diverse populations have complicated the targeting and evaluation of interventions to improve life-course cognitive functioning , especially in LMIC.[8, 9]

Understanding how cognitive functioning tracks across time in diverse settings is an important step towards identifying effective approaches to improve childhood development; however, information from LMIC is limited. Considering that LMIC have the greatest proportion of children at risk and are primary targets for interventions and that the first years of life are the most important period for brain growth and differentiation, it is important to address these gaps in knowledge from LMIC. Following a life-course approach and taking advantage of a long-running cohort from Guatemala, [10] the objective of this study was to assess childhood cognitive developmental trajectories during the first seven years of life, to identify predictors of

For peer review only - http://bmjopen.bmj.com/site/about/guidelines.xhtml

these trajectories, and to estimate the associations of these trajectories with adult cognitive functioning.

METHODS

Longitudinal study

We used data from the INCAP Nutrition Supplementation Trial Longitudinal Study [10]. The study was originally conceived as a trial to test the hypothesis that providing a protein-energy supplement during the preschool years would improve mental development. The specific methods for the original trial have been described extensively; [10-13] briefly, four villages in Guatemala were assigned to receive either a protein-energy gruel (Atole) or a protein-free sugary drink (Fresco). The supplements were provided to all residents in the study villages in a central location twice daily from 1969 through 1977; intakes were recorded for pregnant and lactating women and children under the age of 7 years. In total, 2392 children were enrolled in the original study because they were either younger than seven years at the beginning of the trial or were born during its implementation; hence, participants in this cohort were born between 1962 and 1977 in the study villages.[14] Follow-up studies have been conducted in 1988-99, 2002-04, and 2015-17. [10, 15, 16]

Participant and Public Involvement

Participants and community were not involved in the initial design of the intervention or outcome selection, but over the years the study team provided feedback to the communities about the study and its findings.

For peer review only

Statement of Ethics Approval

This study was approved by Emory University's Institutional Review Board IRB00071041. Parents or legal guardians provided written informed consent for childhood data collection and participants provided written informed consent for each follow-up.

Analytical Sample and Data for this Analysis

We included all participants with at least three measures of cognitive development out of the eight potentially available. This is a conservative approach as latent class growth analysis can handle some participants having as few as one measurement. [17] For the analysis testing associations with adult intelligence and literacy, we excluded participants missing information on these measurements or those without measurements after 18 years of age. Information on sociodemographic predictors such as parents' years of schooling, household socioeconomic status, and distance to the health center was extracted from data collected during the original supplementation study (between 1969 and 1977).

Measures

Cognitive Development Assessments

The cognitive function assessments for infants and preschool children were specifically developed for the study using items from different age-appropriate tests.[18]

Infant battery (administered at child ages 6, 15 and 24 months)-

This set of instruments was administered within a two-week window of infants turning 6, 15, and 24 months. It was developed as a compendium of relevant items from validated infant scales including the Bayley Scales of Infant Development [19], the Gesell and Amatruda's

For peer review only

Developmental Diagnosis [20], the Cattell Infant's Intelligence Scales [21], and Merrill-Palmer Standards of Physical and Mental Growth [22]. The selected items were translated into Spanish and adapted for the Guatemalan context by trained psychologists and psychiatrists. The Composite Infant Score (CIS) summarizes 44 items derived from the previously mentioned scales.

Preschool battery (administered at child ages 3, 4, 5, 6 and 7 years) -

A set of 22 tests was applied within a month of the participants' fifth, sixth and seventh birthday, and a shorter set of 10 tests was applied within a month of children's third and fourth birthdays. The preschool battery measured various dimensions of cognition (e.g. verbal ability, general knowledge, quantitative skills and memory). These scales have previously been analyzed by factor analysis, resulting in a general and a memory factor, which were deemed reliable and valid predictors of later development.[23]

Raven's Progressive Matrices (RPM) –

The RPM was applied during the 1988-1989, 2004-2005, and 2015-2017 data collection waves, when participants were between 11-27, 22-43, and 37-55 years old, respectively. The RPM is a non-verbal test of intellectual functioning and abstract reasoning that includes a series of pattern-matching exercises that get progressively more complex.[24] The RPM traditionally consists of five sets of 12 items each, however only the first three sets were applied, because in our pilot work we found that few participants could process further than that. [14] The score was calculated by summing the number of correct answers. The RPM has good test-retest reliability (intra-class correlation coefficient, 0.87) and internal consistency, as well as construct validity in this Guatemalan population.[14]

For peer review only - http://bmjopen.bmj.com/site/about/guidelines.xhtml

Adult literacy and reading comprehension

InterAmerican Reading Series (Serie Interamericana) –

This test was administered in 2004-2005 and 2015-2017. To complete this test, those who were unable to read a test sentence from the local paper or had not completed primary school, received the pre-screening test questions where scores could range between zero and five. Those who passed the screening test (by answering the five questions correctly) or had at least a secondary school education started with a score of 5 which was then added to the InterAmerican reading and writing series composite score. This approach has been found to have adequate test-retest reliability and internal consistency in this population [25].

Statistical Analysis

Summarizing child cognitive data into trajectories

In order to make the scores comparable over time, we first converted the individual scores for mental development on the CIS at 6, 15 and 24 months, and the preschool series composite score at 3, 4, 5, 6, and 7 years into z-scores relative to the same population and age. We assessed Spearman pairwise correlations between all the available childhood cognitive development measurements during the first seven years and RPM measurements stratified by age. We then used LCGA [17] to cluster participants into developmental trajectories during the first seven years based on the age-adjusted ranked scores. LGCA classifies individuals based on their cognitive development at different ages into classes or trajectories with distinct intercepts and slopes, thereby capturing the heterogeneity within a sample. Because of the way the original study was designed, in order to account for potentially systematically missing information by birth year (supplemental table 1), a sensitivity analysis was conducted by including children with

For peer review only - http://bmjopen.bmj.com/site/about/guidelines.xhtml

at least one CIS and at least one preschool measurement. All LCGA were adjusted for sex and for maternal ID (to account for siblings in the sample). The LCGA analysis was performed using M-Plus (Version 7.0, Los Angeles, CA).

Assessing predictors of child cognitive development trajectories:

We assessed if family-level variables predicted the trajectories using ordinal logistic regression. Models were developed based on theory and available data on the household sociodemographic determinants of child development, which included SES score, parents' education and height, family size (number of people living in the same household), and distance from the supplementation center. All models were adjusted for type of supplement (operationalized as village of residence) and age at exposure to the supplement (first 1000 days vs. not within the first 1000 days). Multiple imputation in SAS 9.4 was conducted to account for item-specific missing values in the predictors. We used PROC MI to generate twenty imputed datasets using fully conditional specification. Ordinal logistic regression was then conducted for each of the twenty imputed datasets and the estimates were pooled using PROC MIANALYZE.

Testing associations between child development measurements, trajectories, and adult outcomes

We conducted a mixed effects linear model using PROC MIXED to test differences in the intercept of all available repeated measurements of adult RPM scores by cognitive development trajectory and by 7-year tertile. For the literacy score, we used the last score recorded and conducted ANOVA tests to assess differences by trajectory and by 7-year tertiles as comparison. Only participants with measurements after 18 years of age were included in these analyses. We tested differences in sociodemographic predictors between included sample and those that had cognitive trajectory information but were lost to follow up due to missing adult measurements.

For peer review only - http://bmjopen.bmj.com/site/about/guidelines.xhtml

Data management and analysis were conducted using SAS 9.4 (SAS Institute Inc., Cary, NC, USA) unless otherwise noted.

RESULTS

The sample for the trajectory analysis and early predictors included 927 participants with at least three measurements of cognitive development during childhood (Supplemental Table 1). Sample sizes for testing associations between trajectory membership and adult cognitive functioning varied; in total, 823 participants could be classified into childhood trajectories and had at least one RPM measurement. Twenty-eight participants had their last RPM measurement conducted when they were younger than 18 years and were excluded from the analysis of trajectories and adult cognitive development. The final sample included for this analysis was of 795 participants (Figure 1) and had similar sociodemographic characteristics to the sample lost to adult follow-up (n=132), except because a larger proportion of men were lost to follow up (data not shown).

[Figure 1- Sample flowchart for cognitive development trajectory analysis in the INCAP Nutrition Supplementation Trial Longitudinal Study in Guatemala]

In the LCGA, based on model fit indexes, the sample membership for each trajectory and interpretability of the resulting classes, we identified three trajectories which we labeled low, average, and high (Figure 2). A sensitivity analysis including only children with at least one infant and one preschool measurement (n=577) also resulted in three similar trajectories. Differences among the three trajectories were apparent already at 6 months and became larger as the children grew older.

[Figure 2: Cognitive development trajectories from 6 months to 7 years (84 months) of age (n=927)]

For peer review only - http://bmjopen.bmj.com/site/about/guidelines.xhtml

The weakest Spearman correlations with the adult RPM assessments were with the infant battery at 6 and 15 months and with the preschool battery at 3 years, followed by the infant battery at 2 years and the preschool battery at 4 years. All the correlations between the 5-, 6-, and 7-year measurements, the trajectories, and the adult RPM were in the moderate range. The strongest correlations between childhood assessments and the RPM were with the preschool battery at 6 years, which ranged between 0.40 (at 11-20 and 21-36 years) and 0.43 (at 37-55 years). The 7-year preschool battery had the strongest correlation with the RPM at ages 11-20 years; the trajectories had the weakest correlation with measurements at 11-20 years (0.30) but stronger correlations with the RPM at 21-36 years (0.35) and 37-55 years (0.38). The RPM at 11-20 years had a correlation of 0.54 with the RPM at 37 to 55 years (Table 1).

Participants in the high trajectory belonged to households that, on average, had higher socioeconomic status, had mothers and fathers with more years of schooling, and had taller mothers, relative to those in the average trajectory. Conversely, the low trajectory had, on average, lower household socioeconomic status, their parents had fewer years of schooling and their mothers were shorter than those in the average trajectory (Table 2).

In proportional odds cumulative logit models, maternal and paternal education and height, and SES were positively associated with the trajectories, while family size was inversely associated with the trajectories (Table 3). After adjusting for all other covariates, the positive association with maternal schooling, maternal height and household SES remained, as did the inverse association with family size.

Child development trajectories were associated with adult RPM and literacy scores (Table 4). The mean and standard deviation for the individual average of RPM scores was 15.2 and 5.2, respectively; on average, participants in the low childhood cognitive development trajectory

For peer review only - http://bmjopen.bmj.com/site/about/guidelines.xhtml

scored 7 fewer points on the RPM compared to those in the high trajectory (a difference of 1.3 standard deviations of the mean). The mean and standard deviation scores for the latest literacy scores were 49.8 and 28.6, respectively. The mean difference between the low and high trajectory for literacy scores was 29.9 (which represents 1.0 standard deviation of the mean). The 7-year cognitive development score tertiles were also associated with RPM and literacy scores in adulthood. Those in the highest 7-year tertile scored on average 5.9 points higher on the RPM and 25.9 points higher on the literacy test compared to those in the lowest tertile.

For peer review only

DISCUSSION

The primary aim of this study was to use a life-course approach to assess child development longitudinally in a longstanding cohort from Guatemala, which has 50 years of follow-up, and, in line with this approach, to identify predictors of early development and correlations with adult non-verbal intelligence and literacy. To our knowledge, this is the first study to assess life-course measures of cognition from early life through adulthood in a LMIC using a developmental trajectory approach, assessing relationships over time.

We identified three trajectories of childhood cognitive development through age seven years that diverge early and then remain parallel through the early school years. Consistent with this finding, early factors such as household SES and family size at birth, as well as maternal years of schooling and height were associated with trajectory membership, independently of other sociodemographic variables. These findings support early life as a critical period and highlight the importance of holistic interventions that address the complex interplay between sociodemographic factors, which ultimately can determine lifelong health advantages or

For peer review only - http://bmjopen.bmj.com/site/about/guidelines.xhtml

vulnerabilities [26]. They also highlight the importance of investing in maternal education and nutrition within a context of a transitioning society to break the inter-generational transmission of inequality.

Results from this analysis, besides supporting early life as a critical period for the development of life-course cognition, also show a cumulative process where single time-point measurements at older ages (5-7 years) have stronger correlations with adult outcomes when compared to measurements conducted during infancy or early childhood (<4 years). The trajectory approach also provided more weight to the information from measurements conducted later in childhood. Taken together, results from this study highlight the importance of child cognitive development as a potential pathway linking early exposures and adult outcomes. This is important for the design and assessment of interventions and programs aiming to improve life-course development, health, human capital, as well as economic development.

We found mid-size correlations of 0.38 between cognitive development trajectories through seven years and non-verbal intelligence measured after 37 years of age. The trajectories created using repeated measurements of cognitive development by different instruments performed similarly to a single time point measurement at age 7 years of cognitive functioning in predicting adult intelligence and literacy in this sample from Guatemala. The use of trajectories has the added advantage of maximizing the sample size, which can decrease bias. The stability of the trajectories decreases the need to conduct multiple tests that increase the risk of chance findings. Similarly, there is no consensus on the best age to assess the impact of interventions on development or to predict later cognitive outcomes as this might vary by setting and population;[1, 27, 28] trajectories allow utilizing all available information rather than relying on a single cross-sectional measurement. Another advantage of using LCGA was that we were able

For peer review only - http://bmjopen.bmj.com/site/about/guidelines.xhtml

to interpret the trajectories and make qualitative inferences about the timing and processes of early cognition within sub-categories of this sample. For example, as mentioned before, the early separation of the trajectories highlights the importance of timing interventions during the prenatal period or early life.

The analysis of the correlation among the trajectories, individual childhood measurements, and adult outcomes also provided insights into the predictive value of individual time-measurements at different ages, which would not be evident with single measurements. In this instance, we showed higher correlations of individual child development measurements after 5 years with both the trajectories and adult non-verbal intelligence, when compared to earlier measurements. These results could be reflective of methodological challenges assessing development in younger children, [11, 29] as well as of cumulative processes and pathways that eventually lead to adult cognition.

A limitation using trajectories is that it can be costly to collect repeated measurements of cognition, and in some cases single measurements can be the only alternative to assess childhood cognitive function. Similarly, missing data is a limitation of this study. Less than half of the participants of the original study had three or more measurements of cognitive development and, due to the study design, most participants have missing data either at younger or older childhood measurements. However, to account for this, we conducted a sensitivity analysis including only those participants with at least one measurement during infancy and at least one measurement during the preschool period and found similar trajectories. Another limitation with the study design was the large age-span of the cohort participants (i.e., 11 years), which forced us to include large age groups for adult measurements and complicated the study of specific periods during adulthood. A limitation of the LCGA approach is that the researcher selects the number

For peer review only - http://bmjopen.bmj.com/site/about/guidelines.xhtml

of classes. We used model fit statistics and class size to select the number of classes but the process is empirical and not necessarily exempt from bias. Finally, there are additional factors that could have contributed to child cognitive development that were not measured in this study; for example, information on parents' intelligence or early childhood care and stimulation, among others factors, were not available.

Global estimates of the number of children not meeting their full developmental potential are currently based on poverty and stunting.[1, 30] Even though these two factors are associated with early childhood development, the use of proxies to assess cognition is limiting [9], and addressing this limitation is important in order to improve our ability to study the determinants of life-course cognition.[9] Results from this study contribute to further our understanding of cognitive functioning across the lifespan in LMIC. This cognitive development trajectory approach sets the basis for the study of life-course predictors of intelligence, which in turn can improve the design and targeting of interventions in diverse populations.

Funding: This study was funded by the Bill and Melinda Gates Foundation OPP1164115. The funder played no role in the design, implementation or interpretation of this study

For peer review only

Table 1: Spearman pairwise correlations between cognitive development tests from 6 months through adulthood (n=1484 with at least 2 measurements of intelligence)

r(n)[a]		Infant Battery			Preschool Battery						Raven Progressive Matrices		
		6 mo	15 mo	24 mo	3 y	4 y	5 y	6 y	7 y	Trajectories	11-20 y	21-36 y	37-55 y
INFANT	6 mo	*1.00* (397)	0.28 (312)	0.14 (293)	0.02 (344)	0.03 (342)	0.11 (277)	0.03 (180)	-0.11 (88)	0.14 (361)	0.08 (266)	0.11 (289)	0.08 (201)
	15 mo		*1.00* (469)	0.28 (312)	0.09 (425)	0.02 (425)	0.17 (332)	0.05 (222)	0.09 (129)	0.21 (447)	0.04 (325)	0.14 (345)	0.11 (236)
	24 mo			*1.00* (466)	0.36 (431)	0.09 (426)	0.16 (361)	0.28 (256)	0.25 (165)	0.39 (452)	0.11 (296)	0.21 (352)	0.18 (238)
PRESCHOOL	3 y				*1.00* (736)	0.29 (692)	0.29 (570)	0.26 (444)	0.24 (329)	0.45 (691)	0.03 (398)	0.09 (567)	0.03 (388)
	4 y					*1.00* (860)	0.41 (696)	0.33 (567)	0.31 (443)	0.53 (806)	0.13 (414)	0.21 (656)	0.23 (450)
	5 y						*1.00* (833)	0.60 (664)	0.54 (533)	0.71 (793)	0.35 (328)	0.34 (596)	0.32 (443)
	6 y							*1.00* (791)	0.66 (632)	0.77 (663)	0.43 (231)	0.40 (502)	0.40 (440)
	7 y								*1.00* (739)	0.76 (540)	0.45 (140)	0.34 (409)	0.35 (422)
	Trajectories[b]									1.00 (927)	0.30 (409)	0.36 (667)	0.38 (495)
RAVEN	11-20y										*1.00* (1297)	0.57 (753)	0.54 (523)
	21-36y											*1.00* (1452)	0.96 (855)
	37-55y												*1.00* (1163)

[a]Values are Spearman correlations between child z-scores and adult raw raven scores.
[b]The trajectories were coded low=0, average=1 and high=2 for this analysis.

For peer review only - http://bmjopen.bmj.com/site/about/guidelines.xhtml

Table 2: Sociodemographic and childhood characteristics by cognitive development trajectory

	**Low (130)**	**Average (583)**	**High (214)[a]**	**p-value**
Community-level predictor				
Large village (%)	56.1	55.3	55.0	0.97
Household-level predictors at enrollment				
Distance from supplementation center (Km)	2.7 ± 0.1	2.5 ± 0.1	2.4 ± 0.1	0.26
SES Score	-0.5 ± 0.1	-0.2 ± 0.1	0.2 ± 0.1	<0.001
Maternal schooling (years)	1.0 ± 0.1	1.2 ± 0.1	1.8 ± 1.1	<0.001
Paternal schooling (years)	1.3 ± 0.2	1.5 ± 0.1	2.0 ± 0.1	<0.001
Maternal height (cm)	148.0 ± 0.5	148.9 ± 0.2	150.0 ± 0.4	<0.001
Paternal height (cm)	160.1 ± 0.6	160.6 ± 0.3	161.6 ± 0.6	0.12
Family size	6.8 ± 0.2	6.7 ± 0.1	6.5 ± 0.2	0.34
Individual-level predictors				
Male (%)	52.6	51.6	54.2	0.83
Height 36 mo (cm)	83.5 ± 0.4	85.5 ± 0.2	86.7 ± 0.3	<0.001
Weight 36 mo (kg)	11.3 ± 0.2	11.8 ± 0.1	12.0 ±0.1	<0.001

[a]Trajectory analysis was adjusted by sex and age at measurement. Differences among groups were assessed using chi-square test for categorical variables and ANOVA for continuous variables.

For peer review only

Table 3: Proportional odds logistic regression of sociodemographic predictors and cognitive development trajectories (n=927)[a]

	Unadjusted		Adjusted[b]	
	Estimate[a]	95% CI	Estimate[a]	95% CI
SES Score	0.22	(0.13, 0.32)	0.19	(0.09, 0.29)
Family size (# of people)	-0.06	(-0.13, 0.00)	-0.08	(-0.16, -0.01)
Distance from supplementation center (Km)	-0.09	(-0.21, 0.03)	-0.03	(-0.16, 0.09)
Parents' schooling (years)				
Maternal	0.20	(0.12, 0.29)	0.15	(0.06, 0.25)
Paternal	0.12	(0.05, 0.18)	0.07	(0.00, 0.15)
Parents' height (cm)				
Maternal	0.04	(0.02, 0.07)	0.03	(0.01, 0.06)
Paternal	0.03	(0.00, 0.05)	0.01	(-0.02, 0.04)

[a] Estimates represent the proportional log odds of following a higher trajectory per unit increase in the predictor. Multiple imputation was used for item-specific missing values in the covariates. Trajectories were coded as low=0, average=1, high=2;

[b] Adjusted for all other covariates and for supplementation with Atole in the first 1000 days (sex and age at measurement were adjusted during the LCGA and thus were not included in this model).

For peer review only - http://bmjopen.bmj.com/site/about/guidelines.xhtml

Table 4: Associations between cognitive development trajectories, preschool series tertiles at 7 years, and adult literacy and non-verbal intelligence

	Trajectory [d]				7-years scores [e]			
	n	Low	Average	High	n	Low	Medium	High
Last available literacy score [a, c]	776	33.9 ± 2.6	48.6 ± 1.2	63.8 ± 2.0	154	33.7 ± 2.5	43.6 ± 2.1	59.6 ± 2.0
Intercept of all available non-verbal intelligence scores	795	11.4 ± 0.9	14.6 ± 0.53	18.4 ± 0.3	154	11.8 ± 1.2	15.0 ± 0.9	17.7 ± 0.8

[a]Measured by the InterAmerican Series;

[b]Measured by the Raven Progressive Matrices (RPM) test; models were adjusted for age at RPM measurement.

[c]Mean ± standard deviation age at last measurement was 40.7 ± 8.8 years;

[d] Values are means and standard errors, mean differences by trajectory were assessed using mixed models (all p-values <0.001);

[e]Values are means and standard errors by Preschool series 7-year score tertiles, mean differences by tertile were assessed using mixed models (all p-values <0.001).

For peer review only

STATEMENTS

A. Contributorship statement

All authors contributed substantially to the conception of the work, acquisition, analysis and/or interpretation of the data as required by BMJ authorship requirements. IGC participated in the conceptualization, conducted the analysis and drafted the original manuscript; ADG, MFKL, LO participated in the conceptualization, data collection and validation, and analysis; MRZ, RM participated in the design and implementation of the original study and the follow-ups; ADS participated in the conceptualization of the follow-ups and the present analysis, supervised and guided the implementation of this study and the writing of the manuscript; all authors reviewed the manuscript critically for intellectual content. All authors agree to be accountable for all aspects of the work reported in this manuscript.

B. Competing Interests

Authors declare no competing interests

C. Funding

This study was funded by the Bill and Melinda Gates Foundation (#OPP1164115). The funder was not involved in the design or interpretation of the results of this analysis. IGC is funded by the National Heart, Blood and Lung Institute awards HL137338-03S1 and HL126146-02.

D. Data Sharing statement

Data are available from the corresponding author upon written request.

E. Ethics Approval

This study was approved by Emory University's Institutional Review Board IRB00071041. Parents or legal guardians provided written informed consent for childhood data collection and participants provided written informed consent for each follow-up.

REFERENCES

1 Black MM, Walker SP, Fernald LCH, *et al.* Early childhood development coming of age: science through the life course. *The Lancet* 2017;**389**:77-90.

For peer review only - http://bmjopen.bmj.com/site/about/guidelines.xhtml

2 Wraw C, Deary IJ, Gale CR, *et al.* Intelligence in youth and health at age 50. *Intelligence* 2015;**53**:23-32.
3 Jokela M, Elovainio M, Singh-Manoux A, *et al.* IQ, socioeconomic status, and early death: The US National Longitudinal Survey of Youth. *Psychosomatic medicine* 2009;**71**:322-8.
4 Calvin CM, Deary IJ, Fenton C, *et al.* Intelligence in youth and all-cause-mortality: systematic review with meta-analysis. *International journal of epidemiology* 2011;**40**:626-44.
5 McHutchison CA, Backhouse EV, Cvoro V, *et al.* Education, Socioeconomic Status, and Intelligence in Childhood and Stroke Risk in Later Life: A Meta-analysis. *Epidemiology (Cambridge, Mass)* 2017;**28**:608-18.
6 Richards M, Deary IJ. A life course approach to cognitive reserve: a model for cognitive aging and development? *Annals of neurology* 2005;**58**:617-22.
7 Kuh D, Network* tNDoAP. A Life Course Approach to Healthy Aging, Frailty, and Capability. *The Journals of Gerontology: Series A* 2007;**62**:717-21.
8 Sabanathan S, Wills B, Gladstone M. Child development assessment tools in low-income and middle-income countries: how can we use them more appropriately? *Archives of disease in childhood* 2015;**100**:482-8.
9 Richter LM, Black M, Britto P, *et al.* Early childhood development: an imperative for action and measurement at scale. *BMJ global health* 2019;**4**:e001302.
10 Stein AD, Melgar P, Hoddinott J, *et al.* Cohort Profile: the Institute of Nutrition of Central America and Panama (INCAP) Nutrition Trial Cohort Study. *International journal of epidemiology* 2008;**37**:716-20.
11 Hack M, Taylor HG, Drotar D, *et al.* Poor predictive validity of the Bayley Scales of Infant Development for cognitive function of extremely low birth weight children at school age. *Pediatrics* 2005;**116**:333-41.
12 Martorell R, Habicht JP, Rivera JA. History and design of the INCAP longitudinal study (1969-77) and its follow-up (1988-89). *J Nutr* 1995;**125**:1027s-41s.
13 Ramirez-Zea M, Mazariegos M. INCAP Longitudinal Study: 50 Years of History and Legacy. *Food and Nutrition Bulletin* 2020;**41**:S5-S7.
14 Pollitt E, Gorman KS, Engle PL, *et al.* Early Supplementary Feeding and Cognition: Effects over Two Decades. *Monographs of the Society for Research in Child Development* 1993;**58**:i-118.
15 Ford ND, Martorell R, Mehta NK, *et al.* Life-Course Body Mass Index Trajectories Are Predicted by Childhood Socioeconomic Status but Not Exposure to Improved Nutrition during the First 1000 Days after Conception in Guatemalan Adults. *The Journal of nutrition* 2016;**146**:2368-74.
16 Ford ND, Behrman JR, Hoddinott JF, *et al.* Exposure to improved nutrition from conception to age 2 years and adult cardiometabolic disease risk: a modelling study. *The Lancet Global Health* 2018;**6**:e875-e84.
17 Jung T, Wickrama KAS. An Introduction to Latent Class Growth Analysis and Growth Mixture Modeling. *Social and Personality Psychology Compass* 2008;**2**:302-17.
18 Engle PL, Fernandez PD. INCAP studies of malnutrition and cognitive behavior. *Food Nutr Bull* 2010;**31**:83-94.
19 Bayley N. *Bayley scales of infant development*. New York: The Psychological Corporation 1969.

For peer review only - http://bmjopen.bmj.com/site/about/guidelines.xhtml

20 Knobloch H, Stevens FM, Malone AF*, et al. Manual of developmental diagnosis : the administration and interpretation of the revised Gesell and Amatruda developmental and neurologic examination*. Hagerstown, Md.: Harper & Row 1980.
21 Cavanaugh MC, Cohen I, Dunphy D*, et al.* Prediction from the Cattell infant intelligence scale. *J Consult Psychol* 1957;**21**:33-7.
22 Merrill-Palmer Institute., Wilson CA. *The Merrill-Palmer standards of physical and mental growth*. Detroit 1930.
23 Pollitt E, Triana N. Stability, Predictive Validity, and Sensitivity of Mental and Motor Development Scales and Pre-School Cognitive Tests among Low-Income Children in Developing Countries. *Food and Nutrition Bulletin* 1999;**20**:45-52.
24 Raven J, Raven J. Raven Progressive Matrices. *Handbook of nonverbal assessment*. New York, NY, US: Kluwer Academic/Plenum Publishers 2003:223-37.
25 Stein AD, Wang M, DiGirolamo A*, et al.* Nutritional supplementation in early childhood, schooling, and intellectual functioning in adulthood: a prospective study in Guatemala. *Arch Pediatr Adolesc Med* 2008;**162**:612-8.
26 Maggi S, Irwin LJ, Siddiqi A*, et al.* The social determinants of early child development: An overview. *Journal of Paediatrics and Child Health* 2010;**46**:627-35.
27 Grantham-McGregor S, Cheung YB, Cueto S*, et al.* Developmental potential in the first 5 years for children in developing countries. *Lancet* 2007;**369**:60-70.
28 Prado EL, Dewey KG. Nutrition and brain development in early life. *Nutrition Reviews* 2014;**72**:267-84.
29 Aslin RN, Fiser J. Methodological challenges for understanding cognitive development in infants. *Trends in Cognitive Sciences* 2005;**9**:92-8.
30 Lu C, Black MM, Richter LM. Risk of poor development in young children in low-income and middle-income countries: an estimation and analysis at the global, regional, and country level. *The Lancet Global Health* 2016;**4**:e916-e22.

For peer review only

For peer review only - http://bmjopen.bmj.com/site/about/guidelines.xhtml

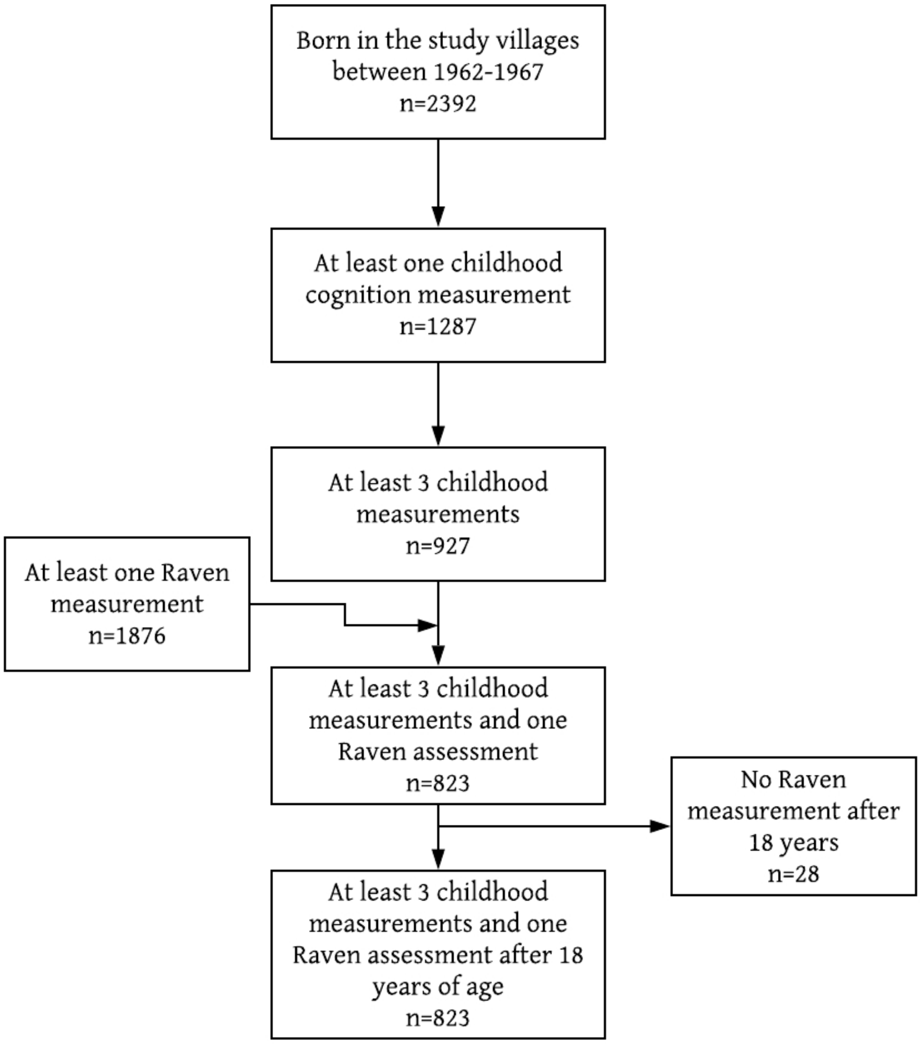

Figure 1- Sample flowchart for cognitive development trajectory analysis in the INCAP Nutrition Supplementation Trial Longitudinal Study in Guatemala

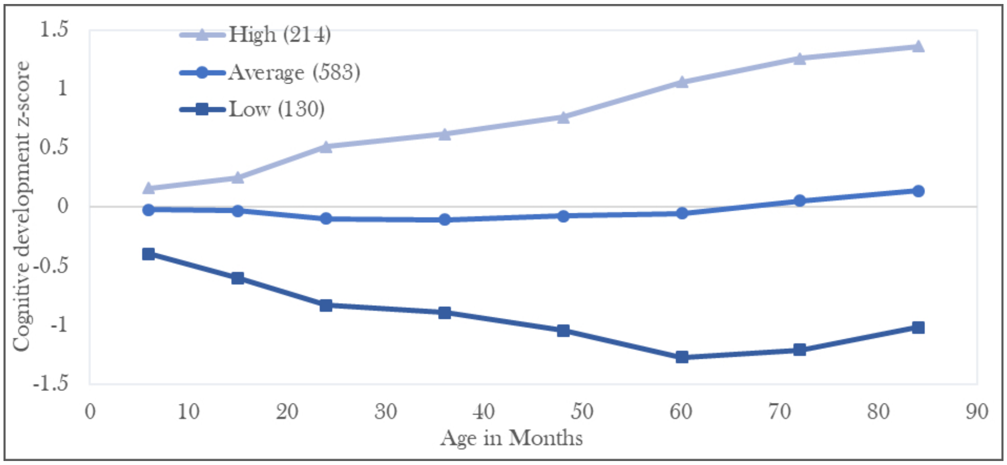

Figure 2: Cognitive development trajectories from 6 months to 7 years (84 months) of age (n=927)

Supplemental Table 1: Sample size of childhood cognitive development and adult Raven Progressive Matrices assessments by birth year in the INCAP Nutrition Supplementation Trial Longitudinal Study (n=2392)

Year of birth	Infant development scales			Preschool Series					≥ 3 child assessments	≥ 1 child assessments	≥ 1 adult assessments	≥ 1 adult and >1 child assessments
	6 months	15 months	24 months	3 years	4 years	5 years	6 years	7 years				
1962	0	0	0	0	0	0	0	47	0	47	60	39
1963	0	0	0	0	0	0	66	86	0	89	80	69
1964	0	0	0	0	0	53	73	78	47	83	63	54
1965	0	0	0	0	63	91	96	97	88	105	97	86
1966	0	0	0	66	98	100	104	101	99	116	113	99
1967	0	1	34	78	92	94	92	96	92	110	112	94
1968	12	44	57	93	95	100	95	94	97	117	114	96
1969	76	84	84	113	118	120	122	122	125	145	146	126
1970	97	95	92	112	113	110	117	18	118	141	143	124
1971	113	116	106	125	128	131	26	0	130	156	146	126
1972	99	114	93	124	129	34	0	0	117	152	168	136
1973	0	15	0	25	24	0	0	0	14	26	133	23
1974	0	0	0	0	0	0	0	0	0	0	148	0
1975	0	0	0	0	0	0	0	0	0	0	133	0
1976	0	0	0	0	0	0	0	0	0	0	135	0
1977	0	0	0	0	0	0	0	0	**0**	0	84	0
**Total**	**397**	**469**	**466**	**736**	**860**	**833**	**791**	**739**	**927**	**1287**	**1875**	**1072**

For peer review only

STROBE Statement—checklist of items that should be included in reports of observational studies

	Item No	Recommendation	Page No
**Title and abstract**	1	(*a*) Indicate the study's design with a commonly used term in the title or the abstract	1
		(*b*) Provide in the abstract an informative and balanced summary of what was done and what was found	1
**Introduction**			
Background/rationale	2	Explain the scientific background and rationale for the investigation being reported	2
Objectives	3	State specific objectives, including any prespecified hypotheses	2
**Methods**			
Study design	4	Present key elements of study design early in the paper	3
Setting	5	Describe the setting, locations, and relevant dates, including periods of recruitment, exposure, follow-up, and data collection	3
Participants	6	(*a*) *Cohort study*—Give the eligibility criteria, and the sources and methods of selection of participants. Describe methods of follow-up *Case-control study*—Give the eligibility criteria, and the sources and methods of case ascertainment and control selection. Give the rationale for the choice of cases and controls *Cross-sectional study*—Give the eligibility criteria, and the sources and methods of selection of participants	3
		(*b*) *Cohort study*—For matched studies, give matching criteria and number of exposed and unexposed *Case-control study*—For matched studies, give matching criteria and the number of controls per case	NA
Variables	7	Clearly define all outcomes, exposures, predictors, potential confounders, and effect modifiers. Give diagnostic criteria, if applicable	4,5
Data sources/ measurement	8*	For each variable of interest, give sources of data and details of methods of assessment (measurement). Describe comparability of assessment methods if there is more than one group	*4,5*
Bias	9	Describe any efforts to address potential sources of bias	6
Study size	10	Explain how the study size was arrived at	4
Quantitative variables	11	Explain how quantitative variables were handled in the analyses. If applicable, describe which groupings were chosen and why	5,6
Statistical methods	12	(*a*) Describe all statistical methods, including those used to control for confounding	5,6
		(*b*) Describe any methods used to examine subgroups and interactions	5,6
		(*c*) Explain how missing data were addressed	5,6
		(*d*) *Cohort study*—If applicable, explain how loss to follow-up was addressed *Case-control study*—If applicable, explain how matching of cases and controls was addressed *Cross-sectional study*—If applicable, describe analytical methods taking account of sampling strategy	NA
		(*e*) Describe any sensitivity analyses	6

Continued on next page

For peer review only - http://bmjopen.bmj.com/site/about/guidelines.xhtml

For peer review only

**Results**			
Participants	13*	(a) Report numbers of individuals at each stage of study—eg numbers potentially eligible, examined for eligibility, confirmed eligible, included in the study, completing follow-up, and analysed	7, Figure 1, Supplemental table 1
		(b) Give reasons for non-participation at each stage	Figure 1
		(c) Consider use of a flow diagram	Figure 1
Descriptive data	14*	(a) Give characteristics of study participants (eg demographic, clinical, social) and information on exposures and potential confounders	7, Table 2
		(b) Indicate number of participants with missing data for each variable of interest	Table 1
		(c) *Cohort study*—Summarise follow-up time (eg, average and total amount)	NA
Outcome data	15*	*Cohort study*—Report numbers of outcome events or summary measures over time	*NA*
		Case-control study—Report numbers in each exposure category, or summary measures of exposure	*NA*
		Cross-sectional study—Report numbers of outcome events or summary measures	*NA*
Main results	16	(*a*) Give unadjusted estimates and, if applicable, confounder-adjusted estimates and their precision (eg, 95% confidence interval). Make clear which confounders were adjusted for and why they were included	8, Table 2, Table 3
		(*b*) Report category boundaries when continuous variables were categorized	NA
		(*c*) If relevant, consider translating estimates of relative risk into absolute risk for a meaningful time period	NA
Other analyses	17	Report other analyses done—eg analyses of subgroups and interactions, and sensitivity analyses	7,8
**Discussion**			
Key results	18	Summarise key results with reference to study objectives	9
Limitations	19	Discuss limitations of the study, taking into account sources of potential bias or imprecision. Discuss both direction and magnitude of any potential bias	11
Interpretation	20	Give a cautious overall interpretation of results considering objectives, limitations, multiplicity of analyses, results from similar studies, and other relevant evidence	9-11
Generalisability	21	Discuss the generalisability (external validity) of the study results	12
**Other information**			
Funding	22	Give the source of funding and the role of the funders for the present study and, if applicable, for the original study on which the present article is based	12

*Give information separately for cases and controls in case-control studies and, if applicable, for exposed and unexposed groups in cohort and cross-sectional studies.

Note: An Explanation and Elaboration article discusses each checklist item and gives methodological background and published examples of transparent reporting. The STROBE checklist is best used in conjunction with this article (freely available on the Web sites of PLoS Medicine at http://www.plosmedicine.org/, Annals of Internal Medicine at

For peer review only

http://www.annals.org/, and Epidemiology at http://www.epidem.com/). Information on the STROBE Initiative is available at www.strobe-statement.org.

For peer review only

For peer review only - http://bmjopen.bmj.com/site/about/guidelines.xhtml

BMJ Open

Association between early child development trajectories and adult cognitive function in a 50-year longitudinal study in Guatemala

Journal:	*BMJ Open*
Manuscript ID	bmjopen-2020-044966.R3
Article Type:	Original research
Date Submitted by the Author:	03-Jun-2021
Complete List of Authors:	Gonzalez Casanova, Ines; Indiana University Bloomington, Applied Health Science; Emory University,<br>DiGirolamo, Ann ; Georgia State University<br>Kroker-Lobos, Maria; INCAP<br>Ochaeta, Laura; INCAP<br>Ramirez-Zea, Manuel; INCAP<br>Martorell, Reynaldo; Emory University<br>Stein, Aryeh D.; Emory University,
**Primary Subject Heading**:	Mental health
Secondary Subject Heading:	Epidemiology, Paediatrics
Keywords:	EPIDEMIOLOGY, International health services < HEALTH SERVICES ADMINISTRATION & MANAGEMENT, MENTAL HEALTH, Developmental neurology & neurodisability < PAEDIATRICS, PUBLIC HEALTH

SCHOLARONE™
Manuscripts

I, the Submitting Author has the right to grant and does grant on behalf of all authors of the Work (as defined in the below author licence), an exclusive licence and/or a non-exclusive licence for contributions from authors who are: i) UK Crown employees; ii) where BMJ has agreed a CC-BY licence shall apply, and/or iii) in accordance with the terms applicable for US Federal Government officers or employees acting as part of their official duties; on a worldwide, perpetual, irrevocable, royalty-free basis to BMJ Publishing Group Ltd ("BMJ") its licensees and where the relevant Journal is co-owned by BMJ to the co-owners of the Journal, to publish the Work in this journal and any other BMJ products and to exploit all rights, as set out in our licence.

The Submitting Author accepts and understands that any supply made under these terms is made by BMJ to the Submitting Author unless you are acting as an employee on behalf of your employer or a postgraduate student of an affiliated institution which is paying any applicable article publishing charge ("APC") for Open Access articles. Where the Submitting Author wishes to make the Work available on an Open Access basis (and intends to pay the relevant APC), the terms of reuse of such Open Access shall be governed by a Creative Commons licence – details of these licences and which Creative Commons licence will apply to this Work are set out in our licence referred to above.

Other than as permitted in any relevant BMJ Author's Self Archiving Policies, I confirm this Work has not been accepted for publication elsewhere, is not being considered for publication elsewhere and does not duplicate material already published. I confirm all authors consent to publication of this Work and authorise the granting of this licence.

For peer review only

Title: Association between early child development trajectories and adult cognitive function in a 50-year longitudinal study in Guatemala

Authors: Ines Gonzalez-Casanova[1,2], Ann DiGirolamo[3], Maria F. Kroker-Lobos[4], Laura Ochaeta[4], Manuel Ramirez-Zea[4], Reynaldo Martorell[2], Aryeh D. Stein[2]*,

[1.] Department of Applied Health Sciences, Indiana University Bloomington School of Public Health, IN, USA, 2. Hubert Department of Global Health, Rollins School of Public Health, Emory University, Atlanta, GA, USA, 3. Georgia Health Policy Center, Andrew Young School of Policy Studies, Georgia State University, Atlanta, Georgia, USA, 4. INCAP Research Center for the Prevention of Chronic Diseases, Institute of Nutrition of Central America and Panama, Guatemala City, Guatemala

***Corresponding Author**
Aryeh D. Stein, PhD
email: aryeh.stein@emory.edu

For peer review only

Abstract

Objectives: Few studies have used longitudinal data to study the development of cognition over the life course in low- and middle- income countries. The objectives of this study were to assess predictors of cognitive development trajectories from 6 months through 7 years, and if these trajectories predicted adult cognitive function in a birth cohort from Guatemala.

Design: We analyzed data from the INCAP Nutrition Supplementation Trial Longitudinal Study in Guatemala. Cognition was assessed at eight different time-points between 6 months and 7 years. We derived childhood development trajectories using latent class growth analysis. We assessed predictors of the trajectories using ordinal logistic regression, and associations between childhood trajectories and adult non-verbal intelligence and literacy at age 18 to 52 years (mean ± SD =42.7 ± 6.4 years) using mixed models.

Setting: The study was conducted in four Guatemalan villages.

Participants: The study included 927 participants from Guatemala with repeated measurements of cognitive function during the first 7 years of life.

Results: We identified three trajectories of cognitive development (high: n=214, average: n=583, low: n=130). Participants whose mothers were taller [proportional log odds (PO)=0.03 95%CI=(0.01, 0.06)], had more years of schooling [PO=0.15 95%CI = (0.06, 0.25)], or lived in households with higher socioeconomic scores [PO = 0.19 95%CI=(0.09, 0.29)] were more likely to follow higher trajectories. Childhood trajectories predicted adult non-verbal intelligence (high=18.4 ± 0.3, average=14.6 ± 0.53, low=11.4 ± 0.9) and literacy (high=18.4 ± 0.3, average=43.6 ± 2.1, low=33.9 ± 2.6) scores.

Conclusions: In this sample from Guatemala, cognitive development trajectories from 6 months through 7 years were associated with adult non-verbal intelligence and literacy. These findings provide evidence of tracking of cognition over-time in a transitioning low-income setting.

Strengths and limitations of this study

- Repeated measures of cognitive function from 6 months to 55 years of age

For peer review only - http://bmjopen.bmj.com/site/about/guidelines.xhtml

- Birth cohort with 50 years of follow-up from Guatemala, a transitioning country
- Longitudinal analysis using latent class growth analysis
- There was missing information from some participants
- Results are consistent with what has been reported in studies from high-income countries

INTRODUCTION

In low- and middle- income (LMIC) countries, 43% of children younger than five years were considered at risk of poor development in 2010 using stunting and poverty as proxies. [1] Early childhood development and childhood intellectual functioning predict survival, lifelong health, and human capital; [2-5] hence, assuring that children worldwide reach their full cognitive developmental potential is central to the sustainable development agenda. [1]

This has led to a widespread call to implement effective interventions that improve early cognitive development.[1, 6, 7] However, the complex nature of the known predictors of early development and challenges assessing development and intelligence across time in diverse populations have complicated the targeting and evaluation of interventions to improve life-course cognitive functioning , especially in LMIC.[8, 9]

Understanding how cognitive functioning tracks across time in diverse settings is an important step towards identifying effective approaches to improve childhood development; however, information from LMIC is limited. Considering that LMIC have the greatest proportion of children at risk and are primary targets for interventions and that the first years of life are the most important period for brain growth and differentiation, it is important to address these gaps in knowledge from LMIC. Following a life-course approach and taking advantage of a long-running cohort from Guatemala, [10] the objective of this study was to assess childhood cognitive developmental trajectories during the first seven years of life, to identify predictors of

For peer review only - http://bmjopen.bmj.com/site/about/guidelines.xhtml

these trajectories, and to estimate the associations of these trajectories with adult cognitive functioning.

METHODS

Longitudinal study

We used data from the INCAP Nutrition Supplementation Trial Longitudinal Study [10]. The study was originally conceived as a trial to test the hypothesis that providing a protein-energy supplement during the preschool years would improve mental development. The specific methods for the original trial have been described extensively; [10-13] briefly, four villages in Guatemala were assigned to receive either a protein-energy gruel (Atole; 119-163 kcal per serving) or a protein-free sugary drink (Fresco; 59 kcal per serving). The supplements were provided to all residents in the study villages in a central location twice daily from 1969 through 1977; intakes were recorded for pregnant and lactating women and children under the age of 7 years. In total, 2392 children were enrolled in the original study because they were either younger than seven years at the beginning of the trial or were born during its implementation; hence, participants in this cohort were born between 1962 and 1977 in the study villages.[14] Follow-up studies have been conducted in 1988-99, 2002-04, and 2015-17. [10, 15, 16]

Participant and Public Involvement

Participants and community were not involved in the initial design of the intervention or outcome selection, but over the years the study team provided feedback to the communities about the study and its findings.

For peer review only - http://bmjopen.bmj.com/site/about/guidelines.xhtml

Statement of Ethics Approval

This study was approved by Emory University's Institutional Review Board IRB00071041. Parents or legal guardians provided written informed consent for childhood data collection and participants provided written informed consent for each follow-up.

Analytical Sample and Data for this Analysis

We included all participants with at least three measures of cognitive development out of the eight potentially available. This is a conservative approach as latent class growth analysis can handle some participants having as few as one measurement. [17] For the analysis testing associations with adult intelligence and literacy, we excluded participants missing information on these measurements or those without measurements after 18 years of age. Information on sociodemographic predictors such as parents' years of schooling, household socioeconomic status, and distance to the health center was extracted from data collected during the original supplementation study (between 1969 and 1977).

Measures

Cognitive Development Assessments

The cognitive function assessments for infants and preschool children were specifically developed for the study using items from different age-appropriate tests.[18]

Infant battery (administered at child ages 6, 15 and 24 months)-

This set of instruments was administered within a two-week window of infants turning 6, 15, and 24 months. It was developed as a compendium of relevant items from validated infant scales including the Bayley Scales of Infant Development [19], the Gesell and Amatruda's

For peer review only

For peer review only - http://bmjopen.bmj.com/site/about/guidelines.xhtml

Developmental Diagnosis [20], the Cattell Infant's Intelligence Scales [21], and Merrill-Palmer Standards of Physical and Mental Growth [22]. The selected items were translated into Spanish and adapted for the Guatemalan context by trained psychologists and psychiatrists. The Composite Infant Score (CIS) summarizes 44 items derived from the previously mentioned scales.

Preschool battery (administered at child ages 3, 4, 5, 6 and 7 years) -

A set of 22 tests was applied within a month of the participants' fifth, sixth and seventh birthday, and a shorter set of 10 tests was applied within a month of children's third and fourth birthdays. The preschool battery measured various dimensions of cognition (e.g. verbal ability, general knowledge, quantitative skills and memory). These scales have previously been analyzed by factor analysis, resulting in a general and a memory factor, which were deemed reliable and valid predictors of later development.[23]

Raven's Progressive Matrices (RPM) –

The RPM was applied during the 1988-1989, 2004-2005, and 2015-2017 data collection waves, when participants were between 11-27, 22-43, and 37-55 years old, respectively. The RPM is a non-verbal test of intellectual functioning and abstract reasoning that includes a series of pattern-matching exercises that get progressively more complex.[24] The RPM traditionally consists of five sets of 12 items each, however only the first three sets were applied, because in our pilot work we found that few participants could process further than that. [14] The score was calculated by summing the number of correct answers. The RPM has good test-retest reliability (intra-class correlation coefficient, 0.87) and internal consistency, as well as construct validity in this Guatemalan population.[14]

For peer review only - http://bmjopen.bmj.com/site/about/guidelines.xhtml

Adult literacy and reading comprehension

InterAmerican Reading Series (Serie Interamericana) –

This test was administered in 2004-2005 and 2015-2017. To complete this test, those who were unable to read a test sentence from the local paper or had not completed primary school, received the pre-screening test questions where scores could range between zero and five. Those who passed the screening test (by answering the five questions correctly) or had at least a secondary school education started with a score of 5 which was then added to the InterAmerican reading and writing series composite score. This approach has been found to have adequate test-retest reliability and internal consistency in this population [25].

Statistical Analysis

Summarizing child cognitive data into trajectories

In order to make the scores comparable over time, we first converted the individual scores for mental development on the CIS at 6, 15 and 24 months, and the preschool series composite score at 3, 4, 5, 6, and 7 years into z-scores relative to the same population and age. We assessed Spearman pairwise correlations between all the available childhood cognitive development measurements during the first seven years and RPM measurements stratified by age. We then used LCGA [17] to cluster participants into developmental trajectories during the first seven years based on the age-adjusted ranked scores. LGCA classifies individuals based on their cognitive development at different ages into classes or trajectories with distinct intercepts and slopes, thereby capturing the heterogeneity within a sample. Because of the way the original study was designed, in order to account for potentially systematically missing information by birth year (supplemental table 1), a sensitivity analysis was conducted by including children with

For peer review only - http://bmjopen.bmj.com/site/about/guidelines.xhtml

at least one CIS and at least one preschool measurement. All LCGA were adjusted for sex and for maternal ID (to account for siblings in the sample). The LCGA analysis was performed using M-Plus (Version 7.0, Los Angeles, CA).

Assessing predictors of child cognitive development trajectories:

We assessed if family-level variables predicted the trajectories using ordinal logistic regression. Models were developed based on theory and available data on the household sociodemographic determinants of child development, which included SES score, parents' education and height, family size (number of people living in the same household), and distance from the supplementation center. All models were adjusted for type of supplement (operationalized as village of residence) and age at exposure to the supplement (first 1000 days vs. not within the first 1000 days). Multiple imputation in SAS 9.4 was conducted to account for item-specific missing values in the predictors. We used PROC MI to generate twenty imputed datasets using fully conditional specification. Ordinal logistic regression was then conducted for each of the twenty imputed datasets and the estimates were pooled using PROC MIANALYZE.

Testing associations between child development measurements, trajectories, and adult outcomes

We conducted a mixed effects linear model using PROC MIXED to test differences in the intercept of all available repeated measurements of adult RPM scores by cognitive development trajectory and by 7-year tertile. For the literacy score, we used the last score recorded and conducted ANOVA tests to assess differences by trajectory and by 7-year tertiles as comparison. Only participants with measurements after 18 years of age were included in these analyses. We tested differences in sociodemographic predictors between included sample and those that had cognitive trajectory information but were lost to follow up due to missing adult measurements.

For peer review only - http://bmjopen.bmj.com/site/about/guidelines.xhtml

Data management and analysis were conducted using SAS 9.4 (SAS Institute Inc., Cary, NC, USA) unless otherwise noted.

RESULTS

The sample for the trajectory analysis and early predictors included 927 participants with at least three measurements of cognitive development during childhood (Supplemental Table 1). Sample sizes for testing associations between trajectory membership and adult cognitive functioning varied; in total, 823 participants could be classified into childhood trajectories and had at least one RPM measurement. Twenty-eight participants had their last RPM measurement conducted when they were younger than 18 years and were excluded from the analysis of trajectories and adult cognitive development. The final sample included for this analysis was of 795 participants (Figure 1) and had similar sociodemographic characteristics to the sample lost to adult follow-up (n=132), except because a larger proportion of men were lost to follow up (data not shown).

[Figure 1- Sample flowchart for cognitive development trajectory analysis in the INCAP Nutrition Supplementation Trial Longitudinal Study in Guatemala]

In the LCGA, based on model fit indexes, the sample membership for each trajectory and interpretability of the resulting classes, we identified three trajectories which we labeled low, average, and high (Figure 2). A sensitivity analysis including only children with at least one infant and one preschool measurement (n=577) also resulted in three similar trajectories. Differences among the three trajectories were apparent already at 6 months and became larger as the children grew older.

[Figure 2: Cognitive development trajectories from 6 months to 7 years (84 months) of age (n=927)]

For peer review only - http://bmjopen.bmj.com/site/about/guidelines.xhtml

The weakest Spearman correlations with the adult RPM assessments were with the infant battery at 6 and 15 months and with the preschool battery at 3 years, followed by the infant battery at 2 years and the preschool battery at 4 years. All the correlations between the 5-, 6-, and 7-year measurements, the trajectories, and the adult RPM were in the moderate range. The strongest correlations between childhood assessments and the RPM were with the preschool battery at 6 years, which ranged between 0.40 (at 11-20 and 21-36 years) and 0.43 (at 37-55 years). The 7-year preschool battery had the strongest correlation with the RPM at ages 11-20 years; the trajectories had the weakest correlation with measurements at 11-20 years (0.30) but stronger correlations with the RPM at 21-36 years (0.35) and 37-55 years (0.38). The RPM at 11-20 years had a correlation of 0.54 with the RPM at 37 to 55 years (Table 1).

Participants in the high trajectory belonged to households that, on average, had higher socioeconomic status, had mothers and fathers with more years of schooling, and had taller mothers, relative to those in the average trajectory. Conversely, the low trajectory had, on average, lower household socioeconomic status, their parents had fewer years of schooling and their mothers were shorter than those in the average trajectory (Table 2).

In proportional odds cumulative logit models, maternal and paternal education and height, and SES were positively associated with the trajectories, while family size was inversely associated with the trajectories (Table 3). After adjusting for all other covariates, the positive association with maternal schooling, maternal height and household SES remained, as did the inverse association with family size.

Child development trajectories were associated with adult RPM and literacy scores (Table 4). The mean and standard deviation for the individual average of RPM scores was 15.2 and 5.2, respectively; on average, participants in the low childhood cognitive development trajectory

For peer review only - http://bmjopen.bmj.com/site/about/guidelines.xhtml

scored 7 fewer points on the RPM compared to those in the high trajectory (a difference of 1.3 standard deviations of the mean). The mean and standard deviation scores for the latest literacy scores were 49.8 and 28.6, respectively. The mean difference between the low and high trajectory for literacy scores was 29.9 (which represents 1.0 standard deviation of the mean). The 7-year cognitive development score tertiles were also associated with RPM and literacy scores in adulthood. Those in the highest 7-year tertile scored on average 5.9 points higher on the RPM and 25.9 points higher on the literacy test compared to those in the lowest tertile.

DISCUSSION

The primary aim of this study was to use a life-course approach to assess child development longitudinally in a longstanding cohort from Guatemala, which has 50 years of follow-up, and, in line with this approach, to identify predictors of early development and correlations with adult non-verbal intelligence and literacy. To our knowledge, this is the first study to assess life-course measures of cognition from early life through adulthood in a LMIC using a developmental trajectory approach, assessing relationships over time.

We identified three trajectories of childhood cognitive development through age seven years that diverge early and then remain parallel through the early school years. Consistent with this finding, early factors such as household SES and family size at birth, as well as maternal years of schooling and height were associated with trajectory membership, independently of other sociodemographic variables. These findings support early life as a critical period and highlight the importance of holistic interventions that address the complex interplay between sociodemographic factors, which ultimately can determine lifelong health advantages or

For peer review only - http://bmjopen.bmj.com/site/about/guidelines.xhtml

vulnerabilities [26]. They also highlight the importance of investing in maternal education and nutrition within a context of a transitioning society to break the inter-generational transmission of inequality.

Results from this analysis, besides supporting early life as a critical period for the development of life-course cognition, also show a cumulative process where single time-point measurements at older ages (5-7 years) have stronger correlations with adult outcomes when compared to measurements conducted during infancy or early childhood (<4 years). The trajectory approach also provided more weight to the information from measurements conducted later in childhood. Taken together, results from this study highlight the importance of child cognitive development as a potential pathway linking early exposures and adult outcomes. This is important for the design and assessment of interventions and programs aiming to improve life-course development, health, human capital, as well as economic development.

We found mid-size correlations of 0.38 between cognitive development trajectories through seven years and non-verbal intelligence measured after 37 years of age. The trajectories created using repeated measurements of cognitive development by different instruments performed similarly to a single time point measurement at age 7 years of cognitive functioning in predicting adult intelligence and literacy in this sample from Guatemala. The use of trajectories has the added advantage of maximizing the sample size, which can decrease bias. The stability of the trajectories decreases the need to conduct multiple tests that increase the risk of chance findings. Similarly, there is no consensus on the best age to assess the impact of interventions on development or to predict later cognitive outcomes as this might vary by setting and population;[1, 27, 28] trajectories allow utilizing all available information rather than relying on a single cross-sectional measurement. Another advantage of using LCGA was that we were able

For peer review only - http://bmjopen.bmj.com/site/about/guidelines.xhtml

to interpret the trajectories and make qualitative inferences about the timing and processes of early cognition within sub-categories of this sample. For example, as mentioned before, the early separation of the trajectories highlights the importance of timing interventions during the prenatal period or early life.

The analysis of the correlation among the trajectories, individual childhood measurements, and adult outcomes also provided insights into the predictive value of individual time-measurements at different ages, which would not be evident with single measurements. In this instance, we showed higher correlations of individual child development measurements after 5 years with both the trajectories and adult non-verbal intelligence, when compared to earlier measurements. These results could be reflective of methodological challenges assessing development in younger children, [11, 29] as well as of cumulative processes and pathways that eventually lead to adult cognition.

A limitation using trajectories is that it can be costly to collect repeated measurements of cognition, and in some cases single measurements can be the only alternative to assess childhood cognitive function. Similarly, missing data is a limitation of this study. Less than half of the participants of the original study had three or more measurements of cognitive development and, due to the study design, most participants have missing data either at younger or older childhood measurements. However, to account for this, we conducted a sensitivity analysis including only those participants with at least one measurement during infancy and at least one measurement during the preschool period and found similar trajectories. Another limitation with the study design was the large age-span of the cohort participants (i.e., 11 years), which forced us to include large age groups for adult measurements and complicated the study of specific periods during adulthood. A limitation of the LCGA approach is that the researcher selects the number

For peer review only - http://bmjopen.bmj.com/site/about/guidelines.xhtml

of classes. We used model fit statistics and class size to select the number of classes but the process is empirical and not necessarily exempt from bias. Finally, there are additional factors that could have contributed to child cognitive development that were not measured in this study; for example, information on parents' intelligence or early childhood care and stimulation, among others factors, were not available.

Global estimates of the number of children not meeting their full developmental potential are currently based on poverty and stunting.[1, 30] Even though these two factors are associated with early childhood development, the use of proxies to assess cognition is limiting [9], and addressing this limitation is important in order to improve our ability to study the determinants of life-course cognition.[9] Results from this study contribute to further our understanding of cognitive functioning across the lifespan in LMIC. This cognitive development trajectory approach sets the basis for the study of life-course predictors of intelligence, which in turn can improve the design and targeting of interventions in diverse populations.

Funding: This study was funded by the Bill and Melinda Gates Foundation OPP1164115. The funder played no role in the design, implementation or interpretation of this study

For peer review only - http://bmjopen.bmj.com/site/about/guidelines.xhtml

Table 1: Spearman pairwise correlations between cognitive development tests from 6 months through adulthood (n=1484 with at least 2 measurements of intelligence)

	r(n)[a]	Infant Battery 6 mo	Infant Battery 15 mo	Infant Battery 24 mo	Preschool Battery 3 y	Preschool Battery 4 y	Preschool Battery 5 y	Preschool Battery 6 y	Preschool Battery 7 y	Trajectories	Raven Progressive Matrices 11-20 y	Raven Progressive Matrices 21-36 y	Raven Progressive Matrices 37-55 y
INFANT	6 mo	*1.00* (397)	0.28 (312)	0.14 (293)	0.02 (344)	0.03 (342)	0.11 (277)	0.03 (180)	-0.11 (88)	0.14 (361)	0.08 (266)	0.11 (289)	0.08 (201)
	15 mo		*1.00* (469)	0.28 (312)	0.09 (425)	0.02 (425)	0.17 (332)	0.05 (222)	0.09 (129)	0.21 (447)	0.04 (325)	0.14 (345)	0.11 (236)
	24 mo			*1.00* (466)	0.36 (431)	0.09 (426)	0.16 (361)	0.28 (256)	0.25 (165)	0.39 (452)	0.11 (296)	0.21 (352)	0.18 (238)
PRESCHOOL	3 y				*1.00* (736)	0.29 (692)	0.29 (570)	0.26 (444)	0.24 (329)	0.45 (691)	0.03 (398)	0.09 (567)	0.03 (388)
	4 y					*1.00* (860)	0.41 (696)	0.33 (567)	0.31 (443)	0.53 (806)	0.13 (414)	0.21 (656)	0.23 (450)
	5 y						*1.00* (833)	0.60 (664)	0.54 (533)	0.71 (793)	0.35 (328)	0.34 (596)	0.32 (443)
	6 y							*1.00* (791)	0.66 (632)	0.77 (663)	0.43 (231)	0.40 (502)	0.40 (440)
	7 y								*1.00* (739)	0.76 (540)	0.45 (140)	0.34 (409)	0.35 (422)
	Trajectories[b]									1.00 (927)	0.30 (409)	0.36 (667)	0.38 (495)
RAVEN	11-20y										*1.00* (1297)	0.57 (753)	0.54 (523)
	21-36y											*1.00* (1452)	0.96 (855)
	37-55y												*1.00* (1163)

[a]Values are Spearman correlations between child z-scores and adult raw raven scores.
[b]The trajectories were coded low=0, average=1 and high=2 for this analysis.

For peer review only - http://bmjopen.bmj.com/site/about/guidelines.xhtml

Table 2: Sociodemographic and childhood characteristics by cognitive development trajectory

	Low (130)	Average (583)	High (214)[a]	p-value
Community-level predictor				
Large village (%)	56.1	55.3	55.0	0.97
Household-level predictors at enrollment				
Distance from supplementation center (Km)	2.7 ± 0.1	2.5 ± 0.1	2.4 ± 0.1	0.26
SES Score	-0.5 ± 0.1	-0.2 ± 0.1	0.2 ± 0.1	<0.001
Maternal schooling (years)	1.0 ± 0.1	1.2 ± 0.1	1.8 ± 1.1	<0.001
Paternal schooling (years)	1.3 ± 0.2	1.5 ± 0.1	2.0 ± 0.1	<0.001
Maternal height (cm)	148.0 ± 0.5	148.9 ± 0.2	150.0 ± 0.4	<0.001
Paternal height (cm)	160.1 ± 0.6	160.6 ± 0.3	161.6 ± 0.6	0.12
Family size	6.8 ± 0.2	6.7 ± 0.1	6.5 ± 0.2	0.34
Individual-level predictors				
Male (%)	52.6	51.6	54.2	0.83
Height 36 mo (cm)	83.5 ± 0.4	85.5 ± 0.2	86.7 ± 0.3	<0.001
Weight 36 mo (kg)	11.3 ± 0.2	11.8 ± 0.1	12.0 ±0.1	<0.001

[a]Trajectory analysis was adjusted by sex and age at measurement. Differences among groups were assessed using chi-square test for categorical variables and ANOVA for continuous variables.

For peer review only

For peer review only - http://bmjopen.bmj.com/site/about/guidelines.xhtml

Table 3: Proportional odds logistic regression of sociodemographic predictors and cognitive development trajectories (n=927)[a]

	Unadjusted		Adjusted[b]	
	**Estimate[a]**	**95% CI**	**Estimate[a]**	**95% CI**
SES Score	0.22	(0.13, 0.32)	0.19	(0.09, 0.29)
Family size (# of people)	-0.06	(-0.13, 0.00)	-0.08	(-0.16, -0.01)
Distance from supplementation center (Km)	-0.09	(-0.21, 0.03)	-0.03	(-0.16, 0.09)
Parents' schooling (years)				
Maternal	0.20	(0.12, 0.29)	0.15	(0.06, 0.25)
Paternal	0.12	(0.05, 0.18)	0.07	(0.00, 0.15)
Parents' height (cm)				
Maternal	0.04	(0.02, 0.07)	0.03	(0.01, 0.06)
Paternal	0.03	(0.00, 0.05)	0.01	(-0.02, 0.04)

[a] Estimates represent the proportional log odds of following a higher trajectory per unit increase in the predictor. Multiple imputation was used for item-specific missing values in the covariates. Trajectories were coded as low=0, average=1, high=2;

[b] Adjusted for all other covariates and for supplementation with Atole in the first 1000 days (sex and age at measurement were adjusted during the LCGA and thus were not included in this model).

For peer review only

Table 4: Associations between cognitive development trajectories, preschool series tertiles at 7 years, and adult literacy and non-verbal intelligence

	Trajectory [d]				7-years scores [e]			
	n	Low	Average	High	n	Low	Medium	High
Last available literacy score [a, c]	776	33.9 ± 2.6	48.6 ± 1.2	63.8 ± 2.0	154	33.7 ± 2.5	43.6 ± 2.1	59.6 ± 2.0
Intercept of all available non-verbal intelligence scores	795	11.4 ± 0.9	14.6 ± 0.53	18.4 ± 0.3	154	11.8 ± 1.2	15.0 ± 0.9	17.7 ± 0.8

[a]Measured by the InterAmerican Series;

[b]Measured by the Raven Progressive Matrices (RPM) test; models were adjusted for age at RPM measurement.

[c]Mean ± standard deviation age at last measurement was 40.7 ± 8.8 years;

[d] Values are means and standard errors, mean differences by trajectory were assessed using mixed models (all p-values <0.001);

[e]Values are means and standard errors by Preschool series 7-year score tertiles, mean differences by tertile were assessed using mixed models (all p-values <0.001).

For peer review only - http://bmjopen.bmj.com/site/about/guidelines.xhtml

STATEMENTS

A. Contributorship statement

All authors contributed substantially to the conception of the work, acquisition, analysis and/or interpretation of the data as required by BMJ authorship requirements. IGC participated in the conceptualization, conducted the analysis and drafted the original manuscript; ADG, MFKL, LO participated in the conceptualization, data collection and validation, and analysis; MRZ, RM participated in the design and implementation of the original study and the follow-ups; ADS participated in the conceptualization of the follow-ups and the present analysis, supervised and guided the implementation of this study and the writing of the manuscript; all authors reviewed the manuscript critically for intellectual content. All authors agree to be accountable for all aspects of the work reported in this manuscript.

B. Competing Interests

Authors declare no competing interests

C. Funding

This study was funded by the Bill and Melinda Gates Foundation (#OPP1164115). The funder was not involved in the design or interpretation of the results of this analysis. IGC is funded by the National Heart, Blood and Lung Institute awards HL137338-03S1 and HL126146-02.

D. Data Sharing statement

Data are available from the corresponding author upon written request.

E. Ethics Approval

This study was approved by Emory University's Institutional Review Board IRB00071041. Parents or legal guardians provided written informed consent for childhood data collection and participants provided written informed consent for each follow-up.

REFERENCES

1 Black MM, Walker SP, Fernald LCH, *et al.* Early childhood development coming of age: science through the life course. *The Lancet* 2017;**389**:77-90.

For peer review only - http://bmjopen.bmj.com/site/about/guidelines.xhtml

2 Wraw C, Deary IJ, Gale CR*, et al.* Intelligence in youth and health at age 50. *Intelligence* 2015;**53**:23-32.
3 Jokela M, Elovainio M, Singh-Manoux A*, et al.* IQ, socioeconomic status, and early death: The US National Longitudinal Survey of Youth. *Psychosomatic medicine* 2009;**71**:322-8.
4 Calvin CM, Deary IJ, Fenton C*, et al.* Intelligence in youth and all-cause-mortality: systematic review with meta-analysis. *International journal of epidemiology* 2011;**40**:626-44.
5 McHutchison CA, Backhouse EV, Cvoro V*, et al.* Education, Socioeconomic Status, and Intelligence in Childhood and Stroke Risk in Later Life: A Meta-analysis. *Epidemiology (Cambridge, Mass)* 2017;**28**:608-18.
6 Richards M, Deary IJ. A life course approach to cognitive reserve: a model for cognitive aging and development? *Annals of neurology* 2005;**58**:617-22.
7 Kuh D, Network* tNDoAP. A Life Course Approach to Healthy Aging, Frailty, and Capability. *The Journals of Gerontology: Series A* 2007;**62**:717-21.
8 Sabanathan S, Wills B, Gladstone M. Child development assessment tools in low-income and middle-income countries: how can we use them more appropriately? *Archives of disease in childhood* 2015;**100**:482-8.
9 Richter LM, Black M, Britto P*, et al.* Early childhood development: an imperative for action and measurement at scale. *BMJ global health* 2019;**4**:e001302.
10 Stein AD, Melgar P, Hoddinott J*, et al.* Cohort Profile: the Institute of Nutrition of Central America and Panama (INCAP) Nutrition Trial Cohort Study. *International journal of epidemiology* 2008;**37**:716-20.
11 Hack M, Taylor HG, Drotar D*, et al.* Poor predictive validity of the Bayley Scales of Infant Development for cognitive function of extremely low birth weight children at school age. *Pediatrics* 2005;**116**:333-41.
12 Martorell R, Habicht JP, Rivera JA. History and design of the INCAP longitudinal study (1969-77) and its follow-up (1988-89). *J Nutr* 1995;**125**:1027s-41s.
13 Ramirez-Zea M, Mazariegos M. INCAP Longitudinal Study: 50 Years of History and Legacy. *Food and Nutrition Bulletin* 2020;**41**:S5-S7.
14 Pollitt E, Gorman KS, Engle PL*, et al.* Early Supplementary Feeding and Cognition: Effects over Two Decades. *Monographs of the Society for Research in Child Development* 1993;**58**:i-118.
15 Ford ND, Martorell R, Mehta NK*, et al.* Life-Course Body Mass Index Trajectories Are Predicted by Childhood Socioeconomic Status but Not Exposure to Improved Nutrition during the First 1000 Days after Conception in Guatemalan Adults. *The Journal of nutrition* 2016;**146**:2368-74.
16 Ford ND, Behrman JR, Hoddinott JF*, et al.* Exposure to improved nutrition from conception to age 2 years and adult cardiometabolic disease risk: a modelling study. *The Lancet Global Health* 2018;**6**:e875-e84.
17 Jung T, Wickrama KAS. An Introduction to Latent Class Growth Analysis and Growth Mixture Modeling. *Social and Personality Psychology Compass* 2008;**2**:302-17.
18 Engle PL, Fernandez PD. INCAP studies of malnutrition and cognitive behavior. *Food Nutr Bull* 2010;**31**:83-94.
19 Bayley N. *Bayley scales of infant development*. New York: The Psychological Corporation 1969.

For peer review only - http://bmjopen.bmj.com/site/about/guidelines.xhtml

20 Knobloch H, Stevens FM, Malone AF*, et al. Manual of developmental diagnosis : the administration and interpretation of the revised Gesell and Amatruda developmental and neurologic examination*. Hagerstown, Md.: Harper & Row 1980.
21 Cavanaugh MC, Cohen I, Dunphy D*, et al.* Prediction from the Cattell infant intelligence scale. *J Consult Psychol* 1957;**21**:33-7.
22 Merrill-Palmer Institute., Wilson CA. *The Merrill-Palmer standards of physical and mental growth*. Detroit 1930.
23 Pollitt E, Triana N. Stability, Predictive Validity, and Sensitivity of Mental and Motor Development Scales and Pre-School Cognitive Tests among Low-Income Children in Developing Countries. *Food and Nutrition Bulletin* 1999;**20**:45-52.
24 Raven J, Raven J. Raven Progressive Matrices. *Handbook of nonverbal assessment*. New York, NY, US: Kluwer Academic/Plenum Publishers 2003:223-37.
25 Stein AD, Wang M, DiGirolamo A*, et al.* Nutritional supplementation in early childhood, schooling, and intellectual functioning in adulthood: a prospective study in Guatemala. *Arch Pediatr Adolesc Med* 2008;**162**:612-8.
26 Maggi S, Irwin LJ, Siddiqi A*, et al.* The social determinants of early child development: An overview. *Journal of Paediatrics and Child Health* 2010;**46**:627-35.
27 Grantham-McGregor S, Cheung YB, Cueto S*, et al.* Developmental potential in the first 5 years for children in developing countries. *Lancet* 2007;**369**:60-70.
28 Prado EL, Dewey KG. Nutrition and brain development in early life. *Nutrition Reviews* 2014;**72**:267-84.
29 Aslin RN, Fiser J. Methodological challenges for understanding cognitive development in infants. *Trends in Cognitive Sciences* 2005;**9**:92-8.
30 Lu C, Black MM, Richter LM. Risk of poor development in young children in low-income and middle-income countries: an estimation and analysis at the global, regional, and country level. *The Lancet Global Health* 2016;**4**:e916-e22.

For peer review only

For peer review only - http://bmjopen.bmj.com/site/about/guidelines.xhtml

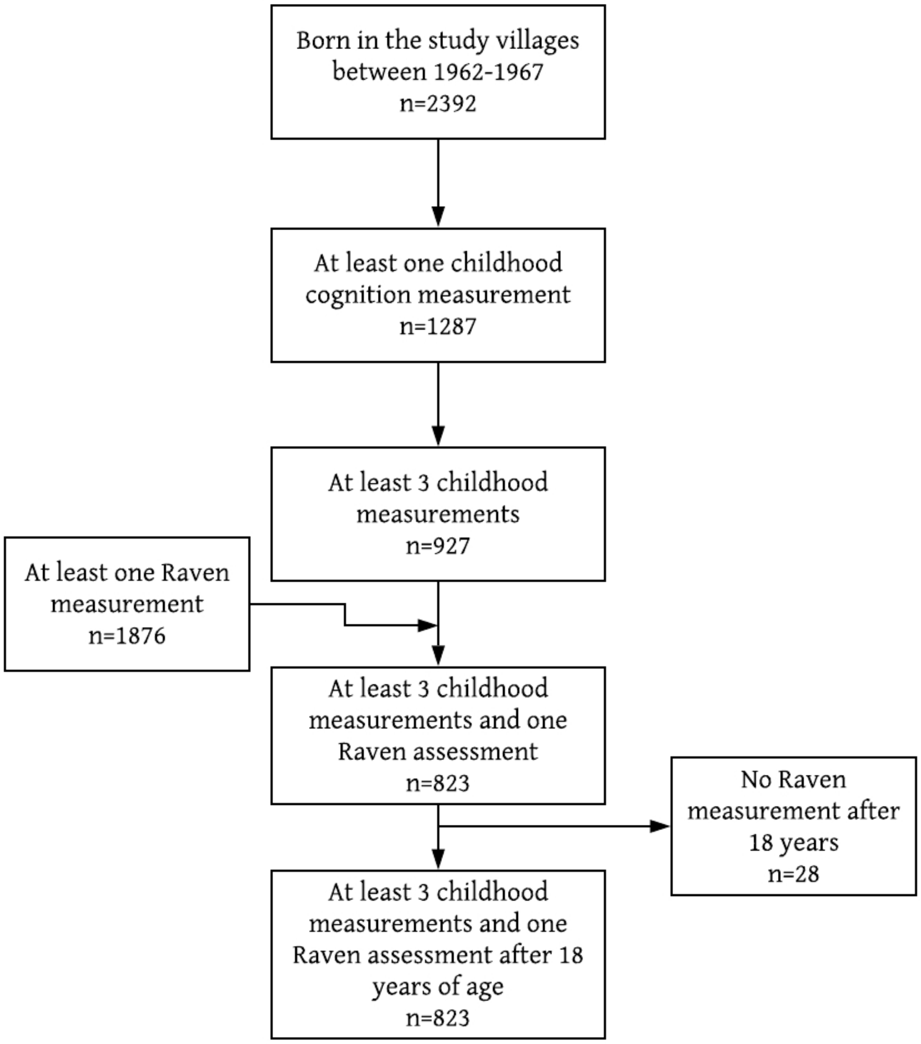

Figure 1- Sample flowchart for cognitive development trajectory analysis in the INCAP Nutrition Supplementation Trial Longitudinal Study in Guatemala

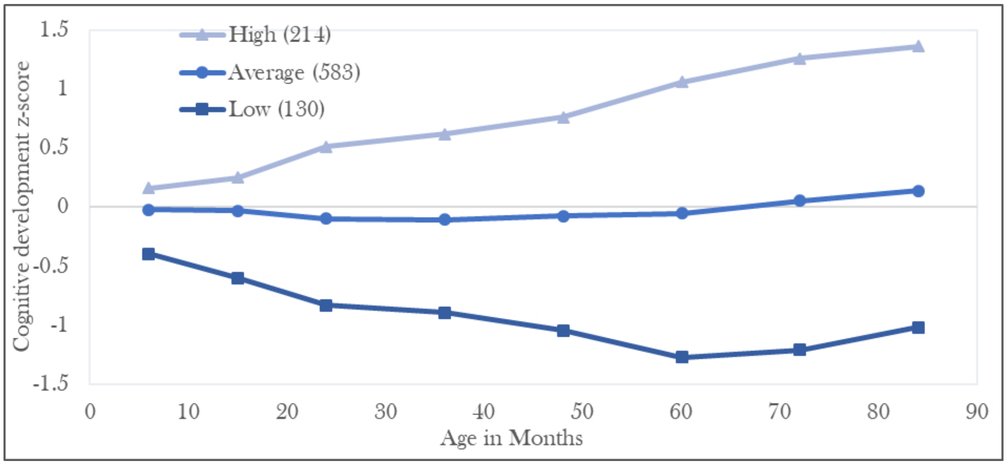

Figure 2: Cognitive development trajectories from 6 months to 7 years (84 months) of age (n=927)

Supplemental Table 1: Sample size of childhood cognitive development and adult Raven Progressive Matrices assessments by birth year in the INCAP Nutrition Supplementation Trial Longitudinal Study (n=2392)

Year of birth	Infant development scales			Preschool Series					≥ 3 child assessments	≥ 1 child assessments	≥ 1 adult assessments	≥ 1 adult and >1 child assessments
	6 months	15 months	24 months	3 years	4 years	5 years	6 years	7 years				
1962	0	0	0	0	0	0	0	47	0	47	60	39
1963	0	0	0	0	0	0	66	86	0	89	80	69
1964	0	0	0	0	0	53	73	78	47	83	63	54
1965	0	0	0	0	63	91	96	97	88	105	97	86
1966	0	0	0	66	98	100	104	101	99	116	113	99
1967	0	1	34	78	92	94	92	96	92	110	112	94
1968	12	44	57	93	95	100	95	94	97	117	114	96
1969	76	84	84	113	118	120	122	122	125	145	146	126
1970	97	95	92	112	113	110	117	18	118	141	143	124
1971	113	116	106	125	128	131	26	0	130	156	146	126
1972	99	114	93	124	129	34	0	0	117	152	168	136
1973	0	15	0	25	24	0	0	0	14	26	133	23
1974	0	0	0	0	0	0	0	0	0	0	148	0
1975	0	0	0	0	0	0	0	0	0	0	133	0
1976	0	0	0	0	0	0	0	0	0	0	135	0
1977	0	0	0	0	0	0	0	0	**0**	0	84	0
**Total**	**397**	**469**	**466**	**736**	**860**	**833**	**791**	**739**	**927**	**1287**	**1875**	**1072**

For peer review only

STROBE Statement—checklist of items that should be included in reports of observational studies

	Item No	Recommendation	Page No
**Title and abstract**	1	(*a*) Indicate the study's design with a commonly used term in the title or the abstract	1
		(*b*) Provide in the abstract an informative and balanced summary of what was done and what was found	1
**Introduction**			
Background/rationale	2	Explain the scientific background and rationale for the investigation being reported	2
Objectives	3	State specific objectives, including any prespecified hypotheses	2
**Methods**			
Study design	4	Present key elements of study design early in the paper	3
Setting	5	Describe the setting, locations, and relevant dates, including periods of recruitment, exposure, follow-up, and data collection	3
Participants	6	(*a*) *Cohort study*—Give the eligibility criteria, and the sources and methods of selection of participants. Describe methods of follow-up<br>*Case-control study*—Give the eligibility criteria, and the sources and methods of case ascertainment and control selection. Give the rationale for the choice of cases and controls<br>*Cross-sectional study*—Give the eligibility criteria, and the sources and methods of selection of participants	3
		(*b*) *Cohort study*—For matched studies, give matching criteria and number of exposed and unexposed<br>*Case-control study*—For matched studies, give matching criteria and the number of controls per case	NA
Variables	7	Clearly define all outcomes, exposures, predictors, potential confounders, and effect modifiers. Give diagnostic criteria, if applicable	4,5
Data sources/ measurement	8*	For each variable of interest, give sources of data and details of methods of assessment (measurement). Describe comparability of assessment methods if there is more than one group	*4,5*
Bias	9	Describe any efforts to address potential sources of bias	6
Study size	10	Explain how the study size was arrived at	4
Quantitative variables	11	Explain how quantitative variables were handled in the analyses. If applicable, describe which groupings were chosen and why	5,6
Statistical methods	12	(*a*) Describe all statistical methods, including those used to control for confounding	5,6
		(*b*) Describe any methods used to examine subgroups and interactions	5,6
		(*c*) Explain how missing data were addressed	5,6
		(*d*) *Cohort study*—If applicable, explain how loss to follow-up was addressed<br>*Case-control study*—If applicable, explain how matching of cases and controls was addressed<br>*Cross-sectional study*—If applicable, describe analytical methods taking account of sampling strategy	NA
		(*e*) Describe any sensitivity analyses	6

Continued on next page

For peer review only - http://bmjopen.bmj.com/site/about/guidelines.xhtml

**Results**			
Participants	13*	(a) Report numbers of individuals at each stage of study—eg numbers potentially eligible, examined for eligibility, confirmed eligible, included in the study, completing follow-up, and analysed	7, Figure 1, Supplemental table 1
		(b) Give reasons for non-participation at each stage	Figure 1
		(c) Consider use of a flow diagram	Figure 1
Descriptive data	14*	(a) Give characteristics of study participants (eg demographic, clinical, social) and information on exposures and potential confounders	7, Table 2
		(b) Indicate number of participants with missing data for each variable of interest	Table 1
		(c) *Cohort study*—Summarise follow-up time (eg, average and total amount)	NA
Outcome data	15*	*Cohort study*—Report numbers of outcome events or summary measures over time	*NA*
		Case-control study—Report numbers in each exposure category, or summary measures of exposure	*NA*
		Cross-sectional study—Report numbers of outcome events or summary measures	*NA*
Main results	16	(*a*) Give unadjusted estimates and, if applicable, confounder-adjusted estimates and their precision (eg, 95% confidence interval). Make clear which confounders were adjusted for and why they were included	8, Table 2, Table 3
		(*b*) Report category boundaries when continuous variables were categorized	NA
		(*c*) If relevant, consider translating estimates of relative risk into absolute risk for a meaningful time period	NA
Other analyses	17	Report other analyses done—eg analyses of subgroups and interactions, and sensitivity analyses	7,8
**Discussion**			
Key results	18	Summarise key results with reference to study objectives	9
Limitations	19	Discuss limitations of the study, taking into account sources of potential bias or imprecision. Discuss both direction and magnitude of any potential bias	11
Interpretation	20	Give a cautious overall interpretation of results considering objectives, limitations, multiplicity of analyses, results from similar studies, and other relevant evidence	9-11
Generalisability	21	Discuss the generalisability (external validity) of the study results	12
**Other information**			
Funding	22	Give the source of funding and the role of the funders for the present study and, if applicable, for the original study on which the present article is based	12

*Give information separately for cases and controls in case-control studies and, if applicable, for exposed and unexposed groups in cohort and cross-sectional studies.

Note: An Explanation and Elaboration article discusses each checklist item and gives methodological background and published examples of transparent reporting. The STROBE checklist is best used in conjunction with this article (freely available on the Web sites of PLoS Medicine at http://www.plosmedicine.org/, Annals of Internal Medicine at

For peer review only

http://www.annals.org/, and Epidemiology at http://www.epidem.com/). Information on the STROBE Initiative is available at www.strobe-statement.org.

For peer review only

For peer review only - http://bmjopen.bmj.com/site/about/guidelines.xhtml
